# Uptake of Cell-Penetrating Peptide RL2 by Human Lung Cancer Cells: Monitoring by Electron Paramagnetic Resonance and Confocal Laser Scanning Microscopy

**DOI:** 10.3390/molecules26185442

**Published:** 2021-09-07

**Authors:** Sergey S. Ovcherenko, Olga A. Chinak, Anton V. Chechushkov, Sergey A. Dobrynin, Igor A. Kirilyuk, Olesya A. Krumkacheva, Vladimir A. Richter, Elena G. Bagryanskaya

**Affiliations:** 1 N.N. Vorozhtsov Novosibirsk Institute of Organic Chemistry SB RAS, 630090 Novosibirsk, Russia; ovcheren@nioch.nsc.ru (S.S.O.); dobrynin@nioch.nsc.ru (S.A.D.); kirilyuk@nioch.nsc.ru (I.A.K.); 2 Institute of Chemical Biology and Fundamental Medicine SB RAS, 630090 Novosibirsk, Russia; chinakolga@gmail.com (O.A.C.); achechushkov@gmail.com (A.V.C.); richter@niboch.nsc.ru (V.A.R.); 3 International Tomography Center SB RAS, 630090 Novosibirsk, Russia; olesya@tomo.nsc.ru

**Keywords:** cell penetrating peptide, electron spin resonance spectroscopy, confocal microscopy

## Abstract

RL2 is a recombinant analogue of a human κ-casein fragment, capable of penetrating cells and inducing apoptosis of cancer cells with no toxicity to normal cells. The exact mechanism of RL2 penetration into cells remains unknown. In this study, we investigated the mechanism of RL2 penetration into human lung cancer A549 cells by a combination of electron paramagnetic resonance (EPR) spectroscopy and confocal laser scanning microscopy. EPR spectra of A549 cells incubated with RL2 (sRL2) spin-labeled by a highly stable 3-carboxy-2,2,5,5-tetraethylpyrrolidine-1-oxyl radical were found to contain three components, with their contributions changing with time. The combined EPR and confocal-microscopy data allowed us to assign these three forms of sRL2 to the spin-labeled protein sticking to the membrane of the cell and endosomes, to the spin-labeled protein in the cell interior, and to spin labeled short peptides formed in the cell because of protein digestion. EPR spectroscopy enabled us to follow the kinetics of transformations between different forms of the spin-labeled protein at a minimal spin concentration (3–16 μM) in the cell. The prospects of applications of spin-labeled cell-penetrating peptides to EPR imaging, DNP, and magnetic resonance imaging are discussed, as is possible research on an intrinsically disordered protein in the cell by pulsed dipolar EPR spectroscopy.

## 1. Introduction

RL2 is a recombinant analogue of a natural peptide, lactaptin, with a molecular weight of 8.6 kDa, that has been isolated from human milk. It has been shown that this peptide is a proteolytic fragment of human k-casein that induces apoptotic death of human breast adenocarcinoma MCF-7 cells in culture [1]. Similarly to lactaptin, its recombinant analogue RL2 (14 kDa) also induces apoptosis of MCF-7 cells as well as other cancer cell types, including A549 cells and is not toxic to nonmalignant human adipose-tissue mesenchymal stem cells (MSCs). It has been reported that RL2 can penetrate the cytoplasm of both MCF-7 cancer cells and nonmalignant adipose-tissue MSCs [2,3,4].

Electron paramagnetic resonance (EPR) spectroscopy has become a powerful tool for studying the structure and dynamics of proteins and nucleic acids [5,6,7]. Continuous-wave EPR spectroscopy at room temperature allows one to study dynamic behavior and molecular motion of a wide variety of biological systems. In electron nuclear double-resonance spectroscopy, electron spin echo envelope modulation, and hyperfine sublevel correlation spectroscopic techniques, electron spin is essentially used as a detector to probe nuclei that are coupled to the unpaired electron spin. Pulsed electron double resonance (PELDOR) [8] or double electron electron resonance (DEER) [9,10] are the most widely used approaches because they enable distance measurements at a nanometer scale between two electron spins introduced into a biomolecule. All the above physical methods imply the use of paramagnetic labels (stable free radicals or metal ions) and a technique of site-specific chemical attachment to a biomolecule in the case when intrinsic paramagnetic centers could not be used. Significant progress has been achieved in the development of these methods in recent years owing to new approaches such as application of special sequences and improvements in hardware—high power amplifiers and arbitrary waveform generators—allowing to substantially increase sensitivity [11,12,13,14,15]. The synthesis of novel spin labels with improved properties, such as long electron spin relaxation time and high stability in biological samples [16], has given an additional impetus to the development of EPR methods in structural biology. Application of such spin labels has made pulsed EPR experiments possible at physiologically relevant temperatures [17,18,19,20] and helped to follow conformational changes in biomolecules inside a cell [21,22,23]. Spin labels based on nitroxides [24], triarylmethyl radicals [18,25,26], and Gd complexes [27,28,29,30,31] have been successfully employed in cell experiments. Nitroxides have clear advantages over alternative spin labels owing to much smaller size (i.e., smaller distortion of a native conformation of a biomolecule) and lower toxicity. Nonetheless, some vital biogenic molecules and enzymatic systems readily reduce nitroxides to diamagnetic compounds, and this situation creates serious obstacles to applications of nitroxide spin labels in biological systems. Notably, high-stability spin probes based on nitroxide radicals [32] are in demand in EPR imaging [33] and magnetic resonance imaging (MRI) as contrast agents [34]. Recently, it has been shown that DEER allows one to study protein structure in cell at nanomolar protein concentration [35].

**Scheme 1:**
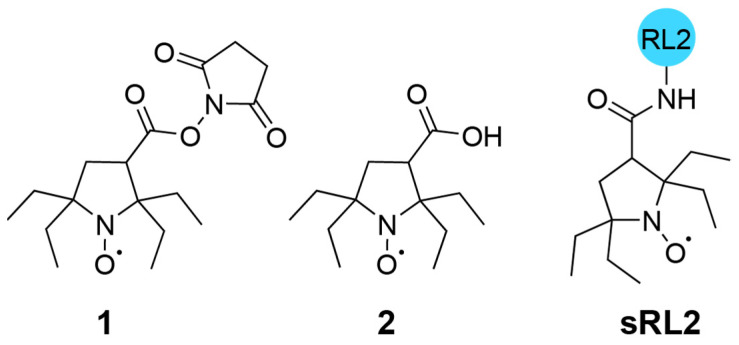
Structural formulas of spin label **1**, nitroxide 2, and spin label 1 attached to RL2 (sRL2).

Bulky substituents (larger than methyl) adjacent to a nitroxide group can retard nitroxide decay in biological systems [24,32,36,37]. So-called sterically shielded nitroxides have much higher resistance to bioreduction as compared to conventional tetramethylnitroxides. For instance, recently, 2,2,5,5-tetraethyl-2,5-dihydropyrrol-1-oxyls were suggested as spin labels for in-cell applications [38]. In the present work, we used the 3-carboxy-2,2,5,5-tetraethylpyrrolidine-1-oxyl N-hydroxysuccinimide ester (1) [39] (see Scheme 1) as a promising spin label for in-cell EPR measurements and tested it on RL2, which can penetrate cells.

**Scheme 2:**
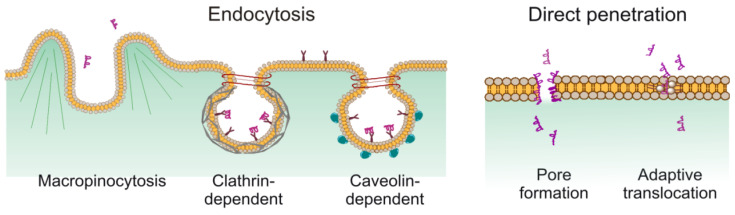
Protein penetration pathways: endocytosis and direct penetration through the plasma membrane.

A number of cell-penetrating peptides (CPPs) are known for their effective penetration and their ability to deliver cargo molecules into a cell. The mechanism of cellular uptake of CPPs is still not well understood, although it is a subject of many studies [40,41]. It is assumed that this uptake can occur via either endocytosis or direct penetration (Scheme 2) [42,43,44]. The main pathway of macromolecule penetration into a cell is endocytosis: adenosine triphosphate (ATP)-dependent transport of macromolecules. It enables recirculation of receptors and lipids, destruction of foreign substances, and uptake of nutrients that cannot passively diffuse through pores in the plasma membrane into a cell. Taken up molecules are internalized by the cell via vesicles. Endocytosis includes phagocytosis (which is more specific for immune cells, e.g., phagocytes), macropinocytosis [45] (uptake of large amounts of fluids driven by rearrangements of filamentous *actin*), and receptor-mediated pinocytosis [46,47] (e.g., clathrin-dependent or caveolin-dependent), which differ in endosome morphology and the proteins involved in the uptake process (Scheme 2).

Various factors influence the penetration of peptides: extracellular concentration of the peptide, the cell line, a combination of the CPP with a high/low molecular weight of the delivered molecule, or a lack thereof. At the same time, all CPP penetration mechanisms, in one way or another, are connected and occur simultaneously, and there may be negative feedback between them.

The mechanism of penetration could be strongly affected by the structure of a peptide. To elucidate the structure–activity relations of RL2, recently we have studied the structural and aggregation features of RL2 by a combination of physicochemical methods: NMR, paramagnetic relaxation enhancement (PRE), EPR, circular dichroism, dynamic light scattering, atomic force microscopy, and a cytotoxic activity assay [48]. It has been shown that RL2 is intrinsically disordered and consists of a mixture of a disulfide-bonded homodimer (RL2_2_) and a conjugate of RL2 with β-mercaptoethanol (BME) via the S–S bond (BME-RL2). The BME conjugate of RL2 is a by-product formed in the process of preparation of the recombinant peptide. RL2 is prone to assembly into large aggregates, and the aggregation increases with ionic strength and pH.

Recently, it has been demonstrated that RL2 labeled by a fluorescent dye penetrates the cell partly by lipid raft–mediated pinocytosis and partly via a pathway alternative to endocytosis [49]. We believe that this nonendocytic pathway may be direct penetration through the plasma membrane. Thus, studying the mechanism of RL2 penetration into the cell may help to elucidate the functions of RL2 and will be useful for the development of new more effective antitumor drugs based on it. Investigation of the behavior of the spin-labeled peptide inside cells can provide more information on the mechanism of RL2 penetration into the cell and on the stability of reduction-resistant nitroxides in the cell. It should be noted that conjugates of nitroxides with biopolymers are considered potential contrast agents for MRI [50,51] or delivery systems for spin probes in EPR imaging or Overhauser enhanced MRI experiments [52,53]. A combination of confocal microscopy and EPR methods may shed light on the mechanism of RL2 penetration because confocal microscopy allows for tracing of the RL2 location in the cell, whereas EPR makes it possible to follow the conversions of RL2 carrying spin label **1** (sRL2) and to detect in real time the moment of protein digestion and detachment of the label.

Here, on the one hand, we studied applicability of spin label **1** to in-cell EPR experiments, and on the other hand, investigated RL2 penetration into human lung cancer A549 cells by EPR spectroscopy and confocal microscopy.

## 2. Materials and Methods

### 2.1. Cell Culture

A549 human lung carcinoma cells (ATCC CCL-185) were grown at 37 °C and 5% CO_2_ in DMEM supplemented with 10% of FBS, 2 mM l-glutamine, 100 U/mL penicillin, 100 μg/mL streptomycin, and 0.25 μg/mL amphotericin B, all from Gibco BRL Co. (Bleiswijk, Netherlands).

During the analysis of spin label **1** and sRL2 internalization, A549 cells were incubated in DMEM (Gibco BRL Co., USA) supplemented with 2 mM l-glutamine, 100 U/mL penicillin, 100 μg/mL streptomycin, and 0.25 μg/mL amphotericin B without FBS. The cells were detached using trypsin for culture procedures (Gibco BRL Co., Bleiswijk, The Netherlands).

### 2.2. Methods

#### 2.2.1. RL2 Expression and Chromatographic Separation of RL2 Dimers and Monomers

RL2 was expressed in the *E. coli* BL21(DE3). RL2 was purified by affinity and ion-exchange chromatography. All buffers contained β-mercaptoethanol. Then RL2 was dialyzed against water. SDS-PAGE analysis showed that RL2 sample consists of a covalent disulfide-bonded homodimer and monomeric peptide (which turned out to be the BME-RL2 adduct) [48].

The RL2_2_ were separated from BME-RL2 by gel-filtration on Superdex 200 (GE Healthcare, Uppsala, Sweden) in running buffer consisting of 0.15 M NaCl, 50 mM NaAc pH 5. Then, low-molecular-weight components were removed using an Amicon Ultra 15 concentrator with a 10 kDa cutoff (Millipore, Burlington, MA, USA).

#### 2.2.2. The Synthesis of the Conjugate of Spin Label **1** with the Recombinant Lactaptin Analogue (sRL2)

A reaction mixture containing the solutions of 1 mM RL2_2_, 5 mM spin label **1**, 55 mM Tris-HCl pH 7, and 15% DMSO was incubated at 25 °C for 7 h (DMSO was added not only because of limited solubility of the spin label in water but also to prevent RL2_2_ aggregation). Considering the propensity of RL2 to aggregate and hydrophobicity of spin label **1**, we extracted spin label **1** from RL2 aggregates by means of guanidine chloride and then dialyzed sRL2 against water. The concentration of the spin label in sRL2 was quantified by EPR spectroscopy, and the concentration of the protein was determined by the Bradford assay. The scheme of labeling of lysine residue using spin label **1** is shown in SI (Figure S1).

#### 2.2.3. Analysis of the Internalization of Spin Label **1** and sRL2 into Cells

Cells were seeded at a density of 8 × 10^5^ cells per culture flask at 24 h prior to the experiment. The culture medium was removed from wells, cells were washed with PBS, and the DMEM medium supplemented with penicillin, streptomycin, amphotericin, l-glutamine, and sRL2 (either 0.5 × 10^−6^ M RL2_2_ and 2 × 10^−6^ M **1** or 0.55 × 10^−5^ M RL2_2_ and 2.2 × 10^−5^ M **1**) dissolved in DMEM was added to the cells and incubated for 1 h at 37 °C. The culture medium was collected for EPR analysis, and the cells were washed twice with PBS and incubated at 37 °C with 300 μL of trypsin to remove the sRL2 localized on the surface of the cell and detach cells from culture flask. The detachment of cells was monitored under a microscope. For trypsin inactivation, DMEM containing 10% of FBS as well as penicillin, streptomycin, amphotericin, and glutamine was added to the cells. The cell suspension was centrifuged for 4 min at 900× *g*. The supernatant was removed, and the cells were washed with PBS and centrifuged under the same conditions; the procedure was carried out twice. After that, 4 μL of the cell pellet was accurately transferred into an EPR capillary tube for measurement.

In the case of the experiment with sodium azide, cells were seeded, incubated for 24 h, and washed with PBS. Then, the DMEM medium with 1% NaN_3_ was added to the cells. After 30 min incubation, the medium was replaced with the DMEM medium supplemented with 1% NaN_3_ and sRL2 (1.34 × 10^−6^ M RL2_2_ and 4.69 × 10^−6^ M **1**). The procedure that followed was performed as described above.

In the experiments with EDTA detachment, cells were seeded as described above, incubated with sRL2 (1.34 × 10^−6^ M RL2_2_ and 4.69 × 10^−6^ M **1**) in DMEM for 10 min and washed with PBS. Then cells were treated with 5 mM EDTA in DMEM for 5 min and gently pipetted. The cells detachment was visually inspected under the microscope. The cell suspension was centrifuged for 4 min at 900× *g*. The cell pellet was washed with PBS twice. After that, 7 μL of the cell pellet was accurately transferred into an EPR capillary tube. The control cells after EDTA detachment were washed with PBS and incubated with 500 μL trypsin to remove the sRL2 from cell surface. After 30 min incubation trypsin was inactivation with DMEM containing 10% FBS, cells were washed and transferred into an EPR capillary tube.

#### 2.2.4. EPR Measurements

X-band EPR spectra were acquired on a commercial Bruker EMX EPR spectrometer (9 GHz, Bruker Spectrospin, Karlsrue, Germany) with a high-sensitivity resonator, Bruker ER4119HS. In experiments at room temperature, the samples were placed into EPR capillary tubes (inner diameter = 0.8 mm, outer diameter = 1 mm). In experiments at 35 °C, the EPR capillary tubes containing the samples were placed into a quartz tube with an inner diameter of 2.0 mm and an outer diameter of 3.0 mm. The temperature was stabilized by a Bruker digital temperature control system, ER 4131VT accessory.

Simulation of the EPR spectra was carried out by means of software package Easy Spin (www.easyspin.org, version 5.2.28) in the slow-motional regime [54,55].

#### 2.2.5. Confocal Microscopy

To determine the intracellular localization of RL2, A549 cells were plated in an μ-dish 35 mm, high (ibiTreat, USA) 18 h prior to the experiment. The cells were washed with PBS and incubated with the DMEM medium supplemented with l-glutamine, antibiotics, and 10^−6^ M fluorescent RL2_2_. After 30 min incubation, the cells were washed twice with PBS and stained for 20 min with LysoSensor Green DND-189, then washed twice and treated with live-cell fluorescent dye Hoechst 33342 in DMEM. In 15 min, it was replaced with DMEM supplemented with 10% of FBS as well as l-glutamine, penicillin, streptomycin, and amphotericin B. The cells were analyzed using the Carl Zeiss LSM 710 laser scanning microscope equipped with a sample-heating module (Carl Zeiss, Jena, Germany). Observations were done using an oil 63× objective. ZEN black edition software (Carl Zeiss, Germany) and CellProfiler software were used in the confocal microscope to visualize images [56].

## 3. Results

### 3.1. The Synthesis of a Conjugate of Spin Label ***1*** with the Recombinant Lactaptin Analogue (sRL2)

**Figure 1:**
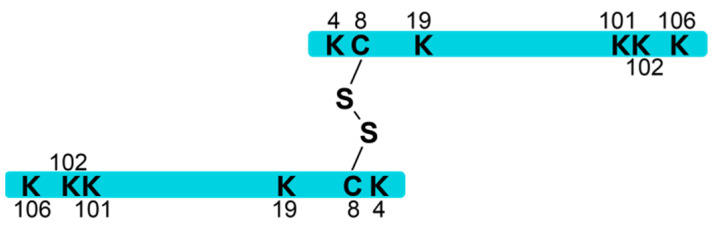
A scheme of RL2_2_. K and C: Lys and Cys, respectively. Numbers indicate the position of the amino acid residues in RL2 sequence.

Reaction of **1** with Lys side chains leads to covalent attachment of the nitroxide (Figure S1). The amino acid sequence of RL2 is shown in Table S1. RL2 contains five Lys residues (Figure 1); two of them (amino acid positions 101 and 102) are adjacent. In this study, the samples of RL2_2_ labeled with spin label **1** are denoted as sRL2. The retention of intact Cys and the presence of only the dimers in the samples was confirmed by nonreducing SDS-PAGE analysis of sRL2 (SI, Figure S2).

The molar ratios were calculated as the ratios of appropriate sRL2 spin concentration determined by EPR to the concentration of the dimer molecules determined by UV spectroscopy. The spin concentration in sRL2 samples was determined via a comparison of the second integral of its EPR spectrum with that of a sample of spin label **1** in an aqueous solution of the same volume and known spin concentration. The average number of spin labels per protein molecule can be regulated by means of an appropriate concentration of spin labels and duration of the spin-labeling procedure. In the case of a large number of spin labels per RL2_2_ molecule (up to 10), the part of the EPR signal corresponding to the obtained sample was strongly broadened (SI, Figures S3 and S4). We assume that this is probably due to the attachment of spin labels to adjacent lysine residues (these spin labels can interact by exchange and dipole–dipole mechanisms); this broadening may complicate subsequent measurements of EPR kinetics. The experimental EPR spectra showed that the broadening was negligible for the ratios of spin labels/RL2_2_ molecules less than 4, because in this case, the probability that both adjacent lysine residues would be labeled simultaneously is much lower (SI Figures S3 and S4).

**Figure 2:**
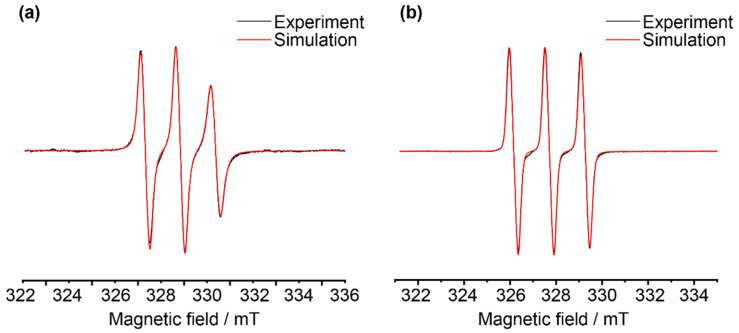
Electron paramagnetic resonance (EPR) spectra and appropriate simulations of (**a**) 0.1 mM **sRL2** (molar ratio of spin label **1** to RL2_2_: 3.5) in an aqueous solution, pH 5.5; (**b**) 0.2 mM radical **2** in 50 mM phosphate-buffered saline (PBS), pH 7.2. The spectra were acquired at 35 °C with the following parameters: microwave frequency (**a**) 9.231393 GHz, (**b**) 9.230876 GHz; (**a**,**b**) microwave power 2.0 mW; (**a**,**b**) sweep time 30.01 s, (**a**,**b**) time constant 20.48 ms, (**a**,**b**) conversion time 29.31 ms, (**a**,**b**) 1024 points, with the number of scans 4 (**a**) and 16 (**b**). In both cases, the external magnetic field was modulated at frequency 100 kHz and modulation amplitude 0.2 mT. Simulations were done in the slow-motional regime [54,55], and parameters of the simulations are given here. g = (2.0091 2.0059 2.0018) (**a**,**b**); hyperfine splitting constants: (**a**) A_N_ = (0.30 0.30 4.00) (mT), and (**b**) A_N_ = (0.30 0.30 4.08) (mT); line width: (**a**) (Gaussian 0.346, Lorentzian 0.0019), (**b**) (Gaussian 0.318, Lorentzian 0.042); Correlation times: (**a**) τ_c_ = 2.77 × 10^−1^ ns, (**b**) τ_c_ = 4.1 × 10^−2^ ns.

Proper simulation of experimental EPR spectra of sRL2 was achieved using the same g- tensor and similar values of N-hyperfine splitting constants (A_hfs_) as those observed for radical **2** (Scheme 1) but with rotation correlation time τ_c_ an order of magnitude higher (Figure 2). Simulation parameter, rotation correlation time τ_c,_ determines the rate of rotational diffusion of the electron spin in a paramagnetic molecule [57]. Higher τ_c_ for sRL2 (τ_c_ = 0.277 ns) reflects lower spin label mobility compared to radical **2** (τ_c_ = 0.041 ns) owing to covalent binding of the small nitroxide molecule to the large RL2_2_ molecule. Tetraethyl nitroxide radicals are known to feature an additional hyperfine splitting constant (hfs) about 0.22 mT on a proton of one of the four ethyl groups and small hfs less than 0.05 mT on other protons [58,59]. For our experimental spectra we compared the simulation with and without this hfs (SI, Figures S5 and S6) and found no substantial difference between them. The only difference was the parameters of linewidth. Thus, in all our simulations,9 this additional hfs was taken into account as increased line width.

### 3.2. Penetration of Nitroxide ***2*** into A549 Cells

A549 cells were incubated for 1 h in the culture medium containing 2 μM nitroxide **2** and washed as described in Methods. The cell suspension and all solutions (the culture medium, PBS, and trypsin solutions) were studied by EPR (SI, Figure S7). No significant changes in the concentration of **2** in the culture medium were observed after the incubation of the cells. No traces of nitroxide **2** were detectable in the cell pellet and in the solutions applied to wash the cells, clearly indicating that nitroxide **2** does not penetrate the cells.

### 3.3. Penetration of sRL2 into A549 Cells

In two independent experiments, A549 cells were incubated in the culture medium containing sRL2 (molar ratio of spin label **1** to RL2_2_: 4) either at 5.5 μM (nitroxide concentration 22 μM) or at 0.5 μM (nitroxide concentration 2 μM). In both cases, after 1 h of cell incubation, the cells were washed according to the procedure described in Methods, and 4 μL of the cell suspension was placed into an EPR capillary tube. EPR spectra of all the solutions applied to wash the cells and the culture medium with dissolved sRL2 before and after the cell incubation are presented in SI, Figures S8–S10. We could not detect an EPR signal in the medium either before or after the cell incubation. Considering that pH of the medium is ~8.5–9.0 (proved by litmus paper), we assume that this finding is related to the aggregation of RL2, which is completely aggregated at pH 7, or to its binding to negatively charged components of the medium, thus resulting in the presence of large particles that are invisible in EPR spectroscopy just as in NMR spectroscopy [48]. To test our assumption and to disrupt the sRL2 aggregates, we added a large excess of HCl into the sample of the medium used for the cell incubation and noticed an EPR signal (SI, Figure S11).

**Figure 3:**
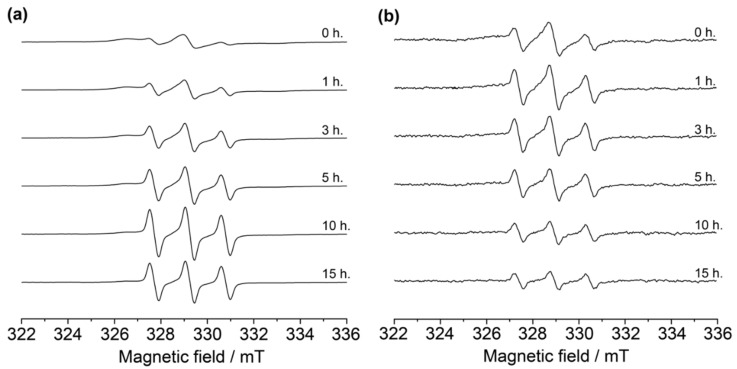
EPR spectra of A549 cells after their 1 h incubation with (**a**) 5.5 μM and (**b**) 0.5 μM sRL2 (molar ratio of spin label 1 to RL2_2_: 4) acquired at different time points after the incubation. The zero time point corresponds to the start of EPR spectra acquisition. Initial spin concentrations observed by EPR at the zero time point for these two samples were 730 μM (**a**) and 60 μM (**b**). The spectra were acquired at 35 °C with the following parameters: microwave frequency (**a**) 9.236816 GHz, (**b**) 9.233912 GHz; (**a**,**b**) microwave power 2.0 mW; conversion time (**a**) 30.89 ms, (**b**) 19.54 ms; sweep time (**a**) 15.82 s, (**b**) 10.00 s; time constant (**a**) 40.96 ms, (**b**) 20.48 ms; (**a**,**b**) 512 points; the number of scans (**a**) 16 or (**b**) 64. In both cases, the external magnetic field was modulated at frequency 100 kHz and modulation amplitude 0.2 mT.

EPR spectra of A549 cells at different time points after the cell incubation with 5.5 μM and 0.5 μM sRL2 are presented in Figure 3a,b, respectively. One can see that the shape of the EPR spectra is changing substantially with time. It is clear that the spectra consist of different EPR spectral components characteristic of different mobility of the spin label, and that the contributions of these components vary with time. At initial time points, the main contribution to the EPR spectra is made by spin labels characterized by a very slow motion, whereas at 15 h, the shape of the EPR spectra is typical for a free nitroxide in an aqueous solution (fast motion). The ratio of different contributions and its dependence on time strongly correlate with the sRL2 initial concentration in the culture medium in which the cells were incubated (see Discussion).

### 3.4. Simulation of EPR Spectra

First, we simulated the experimental spectra by two EPR spectral components (low mobile component 1* and highly mobile component 2*). We can get good agreement between the simulation and the experiment only if we include the decrease of the rotation correlation time for component 2* with the course of the experiment. Such a continuous decrease of the rotation correlation time simultaneously with increase of the weight of the component 2* is hard to explain in the context of penetration of sRL2 into the cell. Therefore, we believe that a more reasonable model is in which the highly mobile spin labels observed in EPR spectra is correspondent to the labels attached to the protein (sRL2) and have similar rotational correlation time as for sRL2 in water or attached to short peptides formed after the protein digestion in lysosomes or proteasomes and have rotational correlation time close to free spin label.

**Figure 4:**
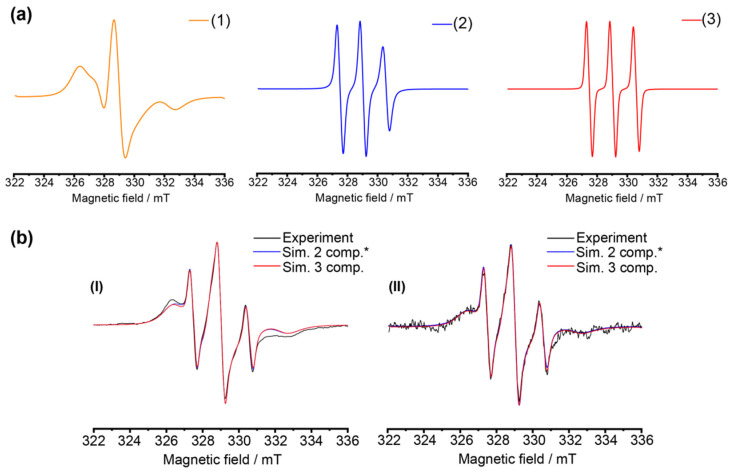
(**a**) Calculated EPR spectral components that were employed to simulate the experimental spectra. The spectra presented are normalized to their central signal height. Spectrum (1) represents the EPR spectral component 1 with calculated rotation correlation time τ_c1_ = 4.77 ns, spectrum (2)—the component 2 with τ_c2_ = 2.95 × 10^−1^ ns and spectrum (3)—the component 3 with τ_c3_ = 4.1 × 10^−2^ ns. Parameters of simulations for all three spectra: g_1_ = g_2_ = g_3_ = [2.0091 2.0059 2.0018]; A_1_ = [0.30 0.30 3.9], mT, A_2_ = [0.30 0.30 4.00], mT, A_3_ = [0.30 0.30 4.08], mT. (**b**) Black: experimental EPR spectra of A549 cells after their incubation with (I) 5.5 μM and (II) 0.5 μM sRL2 (molar ratio of spin label **1** to RL2_2_: 4) acquired at 2.16 h (I) and 0.96 h (II) from the end of the cell incubation. Solid red: simulation of the experimental spectra, which was done by applying the three EPR spectral components with their second integral weight ratios (1):(2):(3) at 0.88:0.07:0.05 (I) and 0.80:0.15:0.05 (II). Short dot blue: simulation of the experimental spectra by only two components (low mobile component 1* and highly mobile component 2*) with the following simulation parameters: g_1*_ = g_2*_ = [2.0091 2.0059 2.0018]; A_1*_ = [0.30 0.30 3.9], mT, A_2*_ = [0.30 0.30 4.05], mT; (I) τ_c1*_ = 4.52 ns, τ_c2*_ = 1.35 × 10^−1^ ns; (II) τ_c1*_ = 4.53 ns, τ_c2*_ = 2.65 × 10^−1^ ns; second integral weight ratios: (1*):(2*) = 0.89:0.11 (I) and (1*):(2*) = 0.85:0.15 (II).

We performed simulation of the obtained EPR spectra of A549 cells after their incubation with sRL2 using simulation parameters obtained for the EPR spectra of sRL2 and radical **2** in aqueous solutions (Figure 4a). We found that all the experimental spectra can be well reproduced by a superposition of three EPR spectral components with different contributions (Figure 4). These three spectral components (Figure 4a) have almost the same anisotropic g-factors and hfs constants: g_1_ = g_2_ = g_3_ = [2.0091 2.0059 2.0018]; A_1_ [A_N_ = (0.30 0.30 3.90) (mT), A_2_ [A_N_ = (0.30 0.30 4.00) (mT), and A_3_ [A_N_ = (0.30 0.30 4.08) (mT)], but different rotational correlation times: τ_c1_ = 4.77 ns, τ_c2_ = 2.95 × 10^−1^ ns, and τ_c3_ = 4.1 × 10^−2^ ns, respectively. One can see that component 2 and component 3 have almost the same simulation parameters (including rotation correlation times) as those of sRL2 and radical **2** (see Figure 2), respectively, in aqueous solutions at the same temperature. Thus, we can assign component 2 to sRL2, which has mobility similar to that found in the aqueous solution, and component 3 to spin labeled short peptides formed in cells because of protein digestion.

Component 1 has the largest contribution to the experimental EPR spectra at initial time points after the cell incubation with sRL2 and refers to spin labels characterized by very slow mobility. It is noteworthy that A_zz_ (3.90 mT) of A_N_ for component 1 is less than A_zz_ (4.00 mT) of A_N_ for component 2, meaning the localization of the spin labels for component 1 in the area with lower polarity in comparison with component 2 [60]. Thus, we can assume that component 1 matches the sRL2 that is localized on the surface of the membrane (endosomal, plasma, or membrane of organelles).

### 3.5. Localization of the Fluorescent RL2_2_ Conjugate in A549 Cells

To refine the nature of the three EPR spectral components, we synthesized fluorescently labeled RL2_2_ and studied its distribution in A549 cells. Briefly, the cells were incubated for 30 min with 10^−6^ M fluorescent RL2_2_ conjugate (fRL2). Then, the cells were treated with blue dye Hoechst 33342, which stains cell nuclei, and LysoSensor Green DND-189: a lipophilic pH indicator that accumulates in acidic organelles with pH below 5.5, e.g., late endosomes and lysosomes. Confocal microscopy was performed on live cells to avoid fixation artifacts such as lysis of endosomes and fRL2 leakage from them. The analysis of confocal microscopy images was performed using the CellProfiler software to quantify the localization of fRL2.

**Figure 5:**
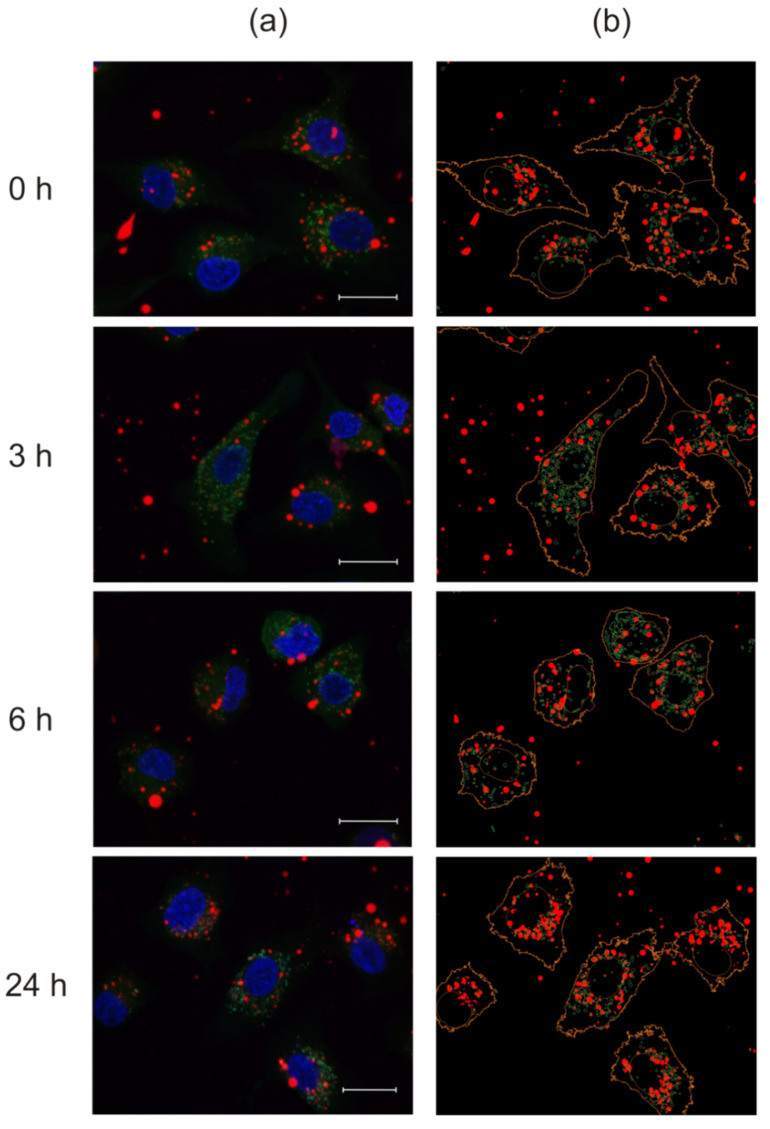
Localization of the fluorescent RL2_2_ conjugate (fRL2) in A549 cells. (**a**) The microscopy analysis. Scale bar, 20 μm. Regions of the intranuclear localization of Hoechst 33342 are blue, regions of fRL2 localization are red, lysosomes and endosomes with pH below 5.5 are green. (**b**) Colocalization analysis (Table S2). Orange lines denote cell edges and nuclei, green lines indicate lysosome and endosome edges, and red signals are fRL2 localization.

Representative confocal images of fRL2 in A549 cells and the processing of the images are shown in Figure 5. The red objects outside the cells are fRL2 attached to the dish surface; they could not be washed off with PBS. As one can see, the red objects inside the cells vary considerably in size. The calculation results on the fRL2 particle number are presented in Table S2. Red signals overlapped with green ones in less than half of the cases. We believe that in the first hours after the cell incubation, a significant portion of fRL2 can be found in early endosomes, which are invisible because of their neutral pH because LysoSensor Green DND-189 accumulates only in acidic organelles. Nonetheless, even at 6 and 24 h from the end of the cell incubation, when endosomes have had enough time to mature, some fRL2 molecules are not in endosomes. This result might be due to endosomal leakage or direct penetration of RL2 bypassing endosomes. This finding is consistent with our earlier study on the influence of different endocytosis inhibitors on fRL2 penetration into cells: RL2 penetrates cells mostly by lipid raft-mediated endocytosis, but none of the endocytosis inhibitors completely suppress its penetration, indicating that RL2 can also penetrate the cells by a mechanism that is an alternative to endocytosis [49]. Thus, we can conclude that RL2 is localized in endosomes and in the cytoplasm of A549 cells after their incubation with RL2.

### 3.6. The Influence of Sodium Azide on the Penetration of sRL2 into A549 Cells

Sodium azide (NaN_3_) is known to inhibit ATP synthesis and thus inhibit endocytosis because endocytosis is an ATP-dependent process [61]. We studied the influence of sodium azide on the penetration of sRL2 into A549 cells. In two independent experiments, cells were incubated in the culture medium containing sRL2 at 1.34 μM (nitroxide concentration 4.69 μM) with and without the addition of 1% NaN_3_. In both cases, after 1 h of cell incubation, the cells were washed according to the procedure described in Methods. EPR spectra of all the solutions that were used to wash out the cells and the culture medium with dissolved sRL2 before and after the cells’ incubation are presented in Figures S12–S14. In the two experiments with and without the addition of NaN_3_, the samples contained either 3 µL of a cell suspension with 8 µL of the medium or 10 µL of a cell suspension with 9 µL of the medium, respectively, to increase the cell viability during the EPR experiments (see Section 3.8).

**Figure 6:**
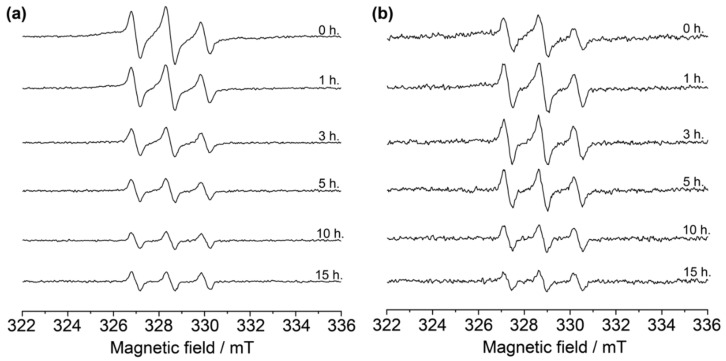
EPR spectra of A549 cells after their 1 h incubation with 1.34 μM sRL2 (molar ratio of spin label **1** to RL2_2_: 3.5) without (**a**) and with (**b**) the addition of 1% NaN_3_, recorded at different time points after the incubation. The zero time point corresponds to the start of EPR spectra acquisition. Initial spin concentrations observed by EPR at the zero time point for these two samples were 87 μM (**a**) and 16 μM (**b**). The spectra were acquired at 35 °C with the following parameters: microwave frequency (**a**) 9.230945 GHz, (**b**) 9.234907 GHz; (**a**,**b**) microwave power 2.0 mW; conversion time (**a**,**b**) 19.56 ms; sweep time (**a**,**b**) 10.01 s; time constant (**a**,**b**) 20.48 ms; (**a**,**b**) 512 points; number of scans (**a**) 32 or (**b**) 64. In both cases, the external magnetic field was modulated at frequency 100 kHz and modulation amplitude 0.2 mT.

**Figure 7:**
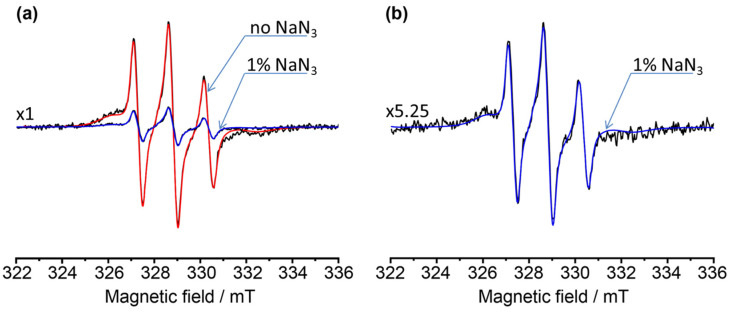
Black: Experimental EPR spectra of A549 cells after their incubation with 1.34 μM sRL2 (molar ratio of spin label **1** to RL2_2_: 3.5) with and without the addition of 1% NaN_3_. (**a**) The comparison of the two experimental spectra acquired at 1.02 h (correspondent to no NaN_3_) and at 0.97 h (correspondent to 1% NaN_3_) from the end of the cell incubation with the same sRL2 concentration. (**b**) The same spectrum correspondent to 1% NaN_3_ presented on (**a**) with a magnification of 5.25 times. Red and Blue: Simulation of the experimental spectra that is carried out by means of the three EPR spectral components discussed above with their second integral weight ratios (1):(2):(3) at 0.69:0.28:0.03 (Red, no NaN_3_) and 0.72:0.23:0.05 (Blue, 1% NaN_3_).

EPR spectra of A549 cells at different time points after the cell incubation with 1.34 μM sRL2 without and with 1% NaN_3_ are presented in Figure 6a,b, respectively. One can see that the addition of sodium azide to the cells incubated with sRL2 leads to a ~5-fold decrease of the total spin concentration and, therefore, to a ~5-fold decrease of the sRL2 concentration in the cells. The spectra in both experiments can be well reproduced by the three EPR spectral components discussed above (for example, see Figure 7).

We detected the presence of component 1 in the experiment with NaN_3_; however, we propose that in this case, endosomes are not formed. We assume that in this case, component 1 can be assigned to sRL2 that is localized on the inner layer of cell membrane. RL2 tends to interact with cell membrane; therefore, it may get into cells through direct penetration (for example, via inverted micelles: a model that is suggested for CPPs) and remain for some time on the inner layer of the cell membrane. Another determination scenario of component 1 is sRL2 that is localized on surfaces of organelles.

### 3.7. Verification of Trypsin Effectivity to Remove sRL2 from Membrane Surface

To prove that trypsin effectively removes sRL2 from the membrane surface we used EDTA to detach cells from the culture flask and performed EPR measurements with and without trypsin incubation (see Section 2.2.3). EPR spectrum obtained after trypsin incubation clearly shows the decrease of immobile fraction in comparison with the same without trypsin incubation (Figure S15). The experiment confirmed that the immobile component 1 detected in our experiments belongs to sRL2 bound to the plasma membrane when cell incubation is short.

### 3.8. Viability of the Cells during the EPR Measurements

We evaluated the viability of A549 cells during all the EPR experiments. The cells incubated with 5.5 or 0.5 μM sRL2 were partially sampled to evaluate their viability in parallel with the EPR experiments. We incubated the cell samples at 37 °C and stained them with trypan blue: a dye that stains dead cells selectively. Counting of dead and live cells revealed that in both cases, ~95% of the cells were alive after 6.5 h (after the end of incubation with sRL2), but 100% were dead after 24 h. Nevertheless, we have previously shown that a half-maximal inhibitory concentration (IC_50_) of RL2 toward A549 cells is 0.39 mg/mL [48]. This means that 48 h incubation of A549 cells with 2.8 × 10^−5^ M RL2 kills 50% of the cells. Consequently, the cell death in the EPR experiments is not a consequence of sRL2 cytotoxicity but rather is due to experimental conditions: the samples for the EPR experiments were cell pellets with a minimal amount of PBS. By contrast, 99.3% of the cells were alive after 10 h and 26.5% after 24 h in the case when PBS was added to the cell pellets after the incubation with 1.34 μM sRL2 and DMEM. In this case, the sample contained 50% volume of the medium. The results were identical in the experiments with and without sodium azide.

## 4. Discussion

In this work, we investigated RL2 penetration into human lung cancer A549 cells by EPR and confocal microscopy and demonstrated the applicability of spin label **1** to in-cell EPR experiments at physiological temperatures. Our data showed that the synthesized spin label **1** can be successfully applied to in-cell EPR experiments with biomolecules which are conducted at 35 °C during more than 15 h. at micromole spin concentrations (Figure 3 and Figure 6). We assume that spin label **1** can also be utilized in EPR imaging, DNP, and MRI.

**Figure 8:**
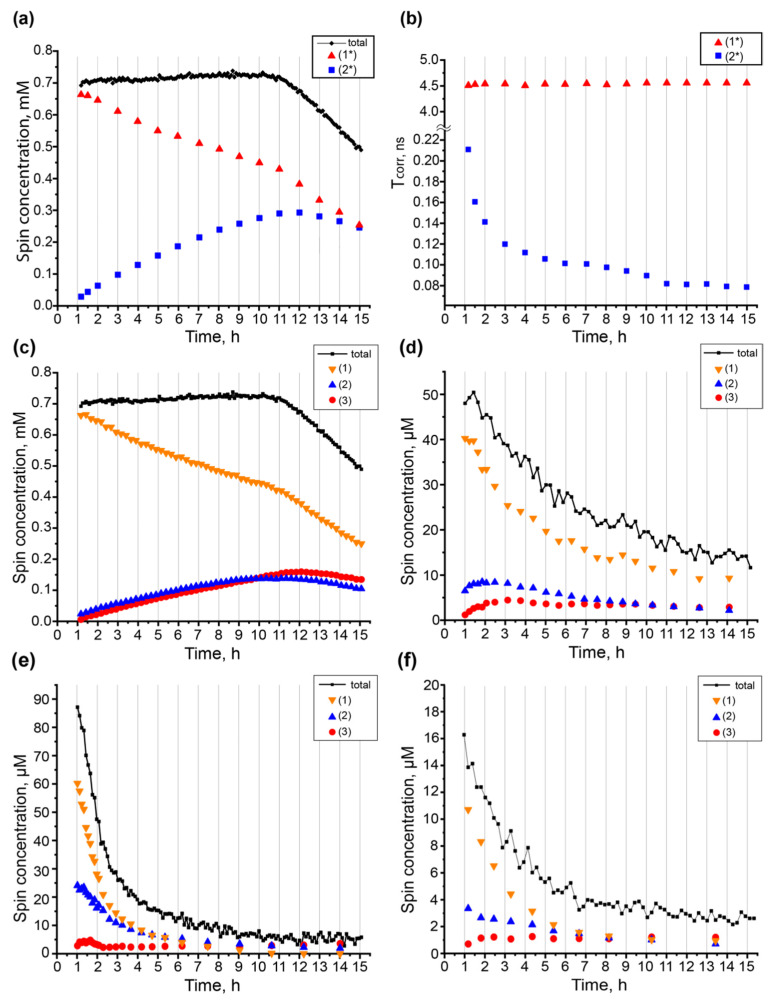
Second-integral decay kinetics and individual-component kinetics of the EPR spectra of A549 cells incubated with (**a**,**c**) 5.5 μM, (**d**) 0.5 μM sRL2 (molar ratio of spin label **1** to RL2_2_: 4) or with 1.34 μM sRL2 (molar ratio of spin label **1** to RL2_2_: 3.5) without (**e**) or with (**f**) the addition of 1% of NaN_3_. (**b**) Time-dependent change of rotational correlation times for EPR spectral components in the case when simulations involve only two components. The time is counted from the end of the cell incubation with sRL2. Black: the total second integral of EPR spectra. (**a**,**b**) Red triangles and blue squares correspond to EPR spectral component 1* and component 2*. (**c**–**f**) Orange inverted triangles, blue triangles, and red circles correspond to EPR spectral component 1, component 2, and component 3, respectively. When the total spin concentration was low, the simulations were performed on the average set of the experimental EPR spectra to attain a better signal/noise ratio and hence better simulation. (**d**) Starting from the 7th point, the simulation was performed on the set of 3, 5, and 7 EPR spectra. (**e**) Starting from the 12th point, the simulation was performed on the set of 3, 5, 7, 9, 15, and 19 EPR spectra. (**f**) Starting from the 1st point, the simulation was performed on the set of 3, 5, 7, 13, and 17 EPR spectra.

We found that all the experimental spectra of the A549 cells incubated with sRL2 can be well reproduced by a superposition of easier two or three EPR spectral components (Figure 4b) with different contributions. We identified these EPR spectral components, corresponding to different spin label mobility. In the case of two components, we have to propose that rotational correlation time correspondent to mobile component (component 2* Figure 8b) is decreasing with time which can be assigned to spin label attached to short oligopeptides formed after digestion of sRL2. The kinetics of the second integral decay of the EPR spectra for low (1*) and highly mobile (2*) components are shown in Figure 8a. As was already mentioned above, it is hard to explain such a continuous decrease of rotational correlation time simultaneously with increasing the weight of component 2* in the context of penetration of sRL2 into the cell.

On the other side our data allows us to assign these three components to sRL2 with mobility similar to that of sRL2 in an aqueous solution (component 2); to spin labeled short peptides or spin labeled amino acid formed in cells due to protein digestion (component 3); to sRL2 that is localized on the surface of some large objects (component 1). As we have previously reported [48] and demonstrated in this work, spin labels in aggregated sRL2 are invisible to EPR. Nonetheless, we can see a low-mobility EPR spectrum (component 1). Therefore, we assigned the low-mobility spectrum to sRL2 bound to some organelles or/and the membrane. In order to refine the origin of component 1 we incubated cells for a short time (10 min) to decrease sRL2 inside cells. Then cells were detached from flask by EDTA which did not remove sRL2 from the cell surface. EPR spectrum of this sample was compared with cells incubated with trypsin for 30 min after EDTA detachment. We showed that sRL2 concentration and share of component 1 are higher in the cells which have not been trypsinized. It means that trypsin digested sRL2 localized on the cell surface. Moreover, sRL2 binding with the cell surface is characterized by very slow mobility (component 1 of EPR spectra).

Therefore, we suppose that in all the EPR experiments, when A549 cells are detached from the flask by trypsin the vast majority of sRL2 is removed from the plasma membrane and the remaining sRL2 with slow mobility of spin labels is located on the inner membrane layer of endosomes. It is in line with confocal microscopy results, some fRL2 signals are colocalized with late endosomes and lysosomes (Figure 5). By contrast, in the EPR experiment with the addition of sodium azide where new endosomes cannot form, we also observed the low-mobility EPR spectrum. We suppose that in this case, the low-mobility component refers to the sRL2 rather situated on surfaces of organelles, which we suppose, are mitochondria, because earlier it was shown that RL2 interact with mitochondrial receptor TOM70 [62] than in the inner membrane layer.

Figure 8 illustrates the second-integral decay kinetics of the EPR spectra obtained for the samples of A549 cells incubated with sRL2 in the four EPR experiments discussed above. The spin concentration in the cells in all the cases was determined by a comparison of the second integral of EPR spectra with that of calibrated samples of spin label **1** in an aqueous solution with the same volumes and temperature. Considering the cell viability assessment during the EPR experiments (see above), EPR experiments were conducted for ≤15 h after the cell incubation. The addition of the culture medium to the cell sample can reduce the signal-to-noise ratio in EPR spectra but improve cell viability, consistently with the data obtained by staining the cells with trypan blue.

We found that the incubation of the cells with RL2 causes protein accumulation in the cells. It is obvious (Figure 8c,d) that EPR measurements of A549 cells incubated with 0.5 or 5.5 μM sRL2 (spin concentrations 2 and 22 μM, respectively) at initial time points showed a spin concentration of 50 and 700 μM, respectively, in the cells; these numbers are ~25- and 30-fold greater than those in the medium used for the cell incubation. On the other hand, the spin concentration observed in A549 cells incubated with 1.34 μM sRL2 (spin concentration 4.69 μM) is 87 μM at an initial time point (see Figure 8e), that is, ~19-fold higher than that in the medium. Thus, A549 cell incubation with micromolar RL2 concentrations leads to RL2 concentrations in the cells two to three dozen times as high as those in the medium.

For the experiment with the highest sRL2 concentration in cells (Figure 8a,c), we observed a plateau in the first 10 h followed by decay during the next 5 h. We believe that the observed plateau is a result of a combination of the spin label reduction in cells and emergence of additional sRL2 from its aggregates, which are not visible to EPR spectroscopy. It is important to note that the higher RL2 concentration in cells leads to greater RL2 aggregation. Accordingly, the plateau is observed only in the EPR experiment with the high sRL2 concentration.

It is known that during endosome maturation, endosomal ATPases pump protons from the cytosol into the endosomes. RL2 contains 10 histidine residues, which themselves can take on pumped protons; thus, a high concentration of RL2 can buffer endosomes. This process is named the “effect of proton sponge” and has been described for histidine-rich CPPs [63]. Such an effect can extend the period of endosome maturation and may sometimes cause osmotic rupture of endosomes. Consequently, the second-integral decay kinetics is slow in the case of the high sRL2 concentration owing to the long maturation of endosomes and fusion with lysosomes. Moreover, the accessibility of the spin label to lysosomal enzymes is low because RL2 is located on a membrane. We assume that the plateau duration during the spin signal decay depends on sRL2 concentration in endosomes.

We noticed that the addition of the endocytosis inhibitor (NaN_3_) leads to a ~5-fold decrease in the total spin concentration (see Figure 8e,f) and hence to a ~5-fold decrease in sRL2 content in the cells. Thus, the main pathway of RL2 penetration into A549 cells is endocytosis, and the main endocytosis pathway is lipid raft–mediated endocytosis, as we have reported earlier [49].

**Scheme 3:**
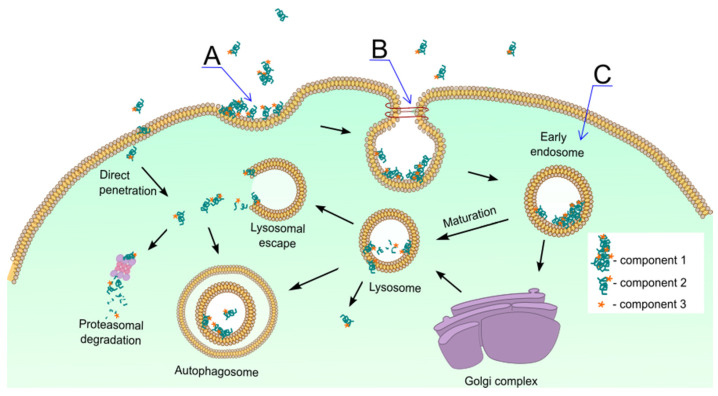
Possible pathways of sRL2 trafficking inside the cell. A—an invagination of the surface of plasma membrane, B—endosome formation, C—formed endosome. Component 1—sRL2 localized on the surface of cell or endosomes, component 2—sRL2 localized inside in the cytoplasm, component 3—spin labeled amino acid formed after protein digestion.

At the first stage of endocytosis, an invagination forms on the surface of the plasma membrane, its curvature increases, and an endosome is formed (Scheme 3A). Then, the neck connecting the plasma membrane with the endosome is pinched off (Scheme 3B), and the early endosome separates from the plasma membrane (Scheme 3C). Cytosolic protons are pumped into early endosomes and they mature to multivesicular bodies. Many smaller vesicles, which are necessary for the sorting of various loads, form inside multivesicular bodies. Some of these small vesicles may bud from the endosomal membrane and deliver the contents directly into the cytosol. Other cargo molecules may be transferred to lysosomes for degradation of the cargo molecules or can be returned to the *trans*-Golgi network for recycling of the cargo molecules back to the plasma membrane.

Spin labeled amino acid molecules, which represent component 3 of the EPR spectra, could be a consequence of proteasomal degradation of sRL2 molecules in the cytoplasm: lysosomal or autophagosomal digestion of sRL2 (Scheme 3). It has been shown that both intrinsically disordered and aggregated proteins located in the cytoplasm are degraded by proteasomes and autophagosomes [64,65,66]. We observed RL2 inside double-membrane vesicles, which we believe are autophagosomes after 6 h of incubation (Figure S16). Earlier, we have shown that RL2 induces autophagy in cancer cells [67,68].

It is more likely that the component 2 corresponds to monomolecular sRL2 molecules inside endosomes or mainly in the cytoplasm. sRL2 could reach the cytoplasm by direct penetration through the plasma membrane, through lysosomal escape, or via a release from multivesicular bodies. The direct penetration is a rapid process, whereas the release from endosomes takes some time. Consequently, the most probable origin of the component 2 in the cytoplasm at the very first moment of EPR spectroscopy (Figure 8) is direct penetration into the cell. The share of component 2 is lower in the cells exposed to the high initial sRL2 concentration (Figure 8c,d). Probably, it is caused by endocytosis stimulation at the high concentration of sRL2, which accumulates on the cell surface. Another possible reason is strong aggregation in the culture medium, thereby leading to the absence of stand-alone sRL2 molecules in endosomes. The weight ratio of component 2 to component 1 is very low and equals 15:80 for the cells incubated with 2 × 10^−6^ M spin label equivalent; therefore, component 2 includes not only the protein that penetrate the cells directly but also stand-alone molecules in endosomes. We should also note that the second form of component 2 could be sRL2 bound on one end to organelles and poses high mobility.

The EPR method alone is not enough to distinguish the localization inside endosomes from the localization inside the cytoplasm. This problem could be solved by means of a pH-sensitive spin label. Unfortunately, until recently, pH-sensitive spin probes have not been very resistant to reduction and could not be used for in-cell EPR experiments; the design of suitable spin probes is underway.

The combination of confocal microscopy and EPR spectroscopy is useful for the research on peptide penetration for the following reasons. On the one hand, EPR allows us to trace in real time the kinetics of a single protein state transformation but does not show the location of the protein in cells. On the other hand, confocal microscopy makes it possible to trace RL2 locations in the cell, but we cannot distinguish the signal of the labeled protein from the residual fluorescent dye label after protein digestion. Taken together, these methods help us to shed light on the complete picture of RL2 penetration.

## 5. Conclusions

In this study, we employed reduction-resistant spin label **1** based on 3-carboxy-2,2,5,5-tetraethylpyrrolidine-1-oxyl (**2**). It is shown that stable spin probe **2** cannot penetrate into the cell, whereas the attachment of this spin label to a human kappa casein fragment, RL2, capable of penetrating the cell allows us to study its stability in human lung adenocarcinoma A549 cells and to follow its transformation inside the cells by EPR spectroscopy. The stability of spin label molecules is very high and makes it possible to investigate the kinetics and changes in the EPR spectra for more than 15 h. It is demonstrated that the shape of the EPR spectra is affected by three contributions: the spin-labeled protein with very low mobility (which mostly sticks to the membrane of endosomes), the spin-labeled protein with mobility similar to that of sRL2 in an aqueous solution, and the spin labeled short peptides formed in the cells owing to protein digestion. EPR helps us to follow the kinetics of different forms of sRL2 and its transformation at micromolar sRL2 concentration in cells.

The results indicate that the EPR approach may be used to investigate the kinetics and mechanisms of penetration of a non-aggregating protein into the cell. CPPs are known for their effective penetration of the cell via direct passage through the plasma membrane or endocytosis and for their ability to deliver cargo molecules into the cell. Unlike electroporation and microinjection, intracellular delivery of cargo molecules using CPP is suitable for in vivo applications. It is possible to increase the spin label-to-protein ratio in order to raise the concentration of the spin label in the cell. We noticed that after the penetration of the spin-labeled protein into the cell, the label gets detached and features narrow lines in the EPR spectrum. This approach can be utilized for gentle and efficient delivery of spin probes during EPR imaging and for the design of a DNP agent. To increase sensitivity, the deuteration of the ethyl group in spin label **1** can ensure narrow lines of free nitroxides and increase EPR imaging sensitivity.

## Data Availability

Not applicable.

## Supplementary Materials

The following are available online, Figure S1: Labeling of lysine residue using spin label 1; Figure S2: SDS-PAGE: first column—marker 4–20% TRIS-glycine SDS-PAGE; second column clean RL2 dimer (RL2_2_); Figure S3: EPR spectra of the samples of sRL2 in aqueous solution with different molar ratios of spin label 1 to RL2_2_; Figure S4: EPR spectra of A549 cells incubated with sRL2 samples with molar ratios of spin label 1 to RL2_2_; Figure S5: Experimental EPR spectra of radical 2 in phosphate-buffered saline and sRL2 in an aqueous solution; Figure S6: Experimental EPR spectrum of A549 cells after their incubation with sRL2; Figure S7: EPR spectra of cell medium with dissolved nitroxide 2 before and after cells incubation; Figure S8: CW EPR spectra of the cell medium with dissolved sRL2; Figure S9: CW EPR spectra of trypsin solutions after their application to wash the cells incubated with high, and low sRL2 concentrations; Figure S10: CW EPR spectra of PBS solutions after their application to wash the cells before and after cells washing by trypsin; Figure S11: CW EPR spectrum of the cell medium with dissolved sRL2 after addition of large excess of HCl; Figure S12: CW EPR spectra of the cell medium with dissolved sRL2; Figure S13: CW EPR spectra of trypsin solutions after their application to wash the cells incubated in the medium with dissolved sRL2; Figure S14: CW EPR spectra of PBS solutions after their application to wash the cells incubated with sRL2; Figure S15: Brief cells incubation with sRL2. Exposure to trypsin; Figure S16: Localization of the fluorescent RL2 conjugate in A549 cells in 6 h after their incubation; Figure S17: Simulation of experimental spectra by two components. Table S1. RL2 sequence in single letter designation. Highlighted letters: (green)—lysine, (blue)—cysteine. Table S2: Colocalization analysis of green and red signals inside A549 cells.

## Sample Availability

Sample of the compound **1** is available from the authors.
