# Supplementary material for: Uptake of Cell-Penetrating Peptide RL2 by Human Lung Cancer Cells: Monitoring by Electron Paramagnetic Resonance and Confocal Laser Scanning Microscopy
Source: Molecules. 2021 Sep 7;26(18):5442. doi: 10.3390/molecules26185442 (PMC8470091; doi:10.3390/molecules26185442)
Supplement: Supplementary file 1 [file molecules-26-05442-s001.zip › molecules-1329338-supplementary.pdf]

Uptake of cell-penetrating peptide RL2 by human lung cancer cells: monitoring by Electron Paramagnetic Resonance and confocal laser scanning microscopy

S.S. Ovcherenko[1], O.A. Chinak[2], A.V. Chechushkov[2], S.A. Dobrynin,[1] I.A. Kirilyuk[1], O.A. Krumkacheva[3], V.A. Richter[2], E.G. Bagryanskaya[1]*

[1] N.N. Vorozhtsov Novosibirsk Institute of Organic Chemistry SB RAS, Novosibirsk 630090, Russia,
[2] Institute of Chemical Biology and Fundamental Medicine SB RAS, Novosibirsk 630090, Russia
[3]International Tomography Center SB RAS, Novosibirsk 630090, Russia

Electronic Supplementary Information

Table of contents

RL2 sequence. SI2

Spin labeling of RL2. SI2

EPR spectra of the samples of sRL2 in aqueous solution with different molar ratios of spin label **1** to $RL2_2$ SI3

EPR spectra of A549 cells incubated with sRL2 samples with molar ratios of spin label **1** to $RL2_2$ SI3

Simulations of EPR spectra with and without hfs A_H SI4-SI5

EPR spectra of cultural media and solutions applied to wash A549 cells in the experiment of the cells incubation with nitroxide **2.** SI6

EPR spectra of cultural media and solutions applied to wash A549 cells in the experiments of the cells incubation with sRL2. SI7-SI8

EPR spectra of cultural media and solutions applied to wash A549 cells in the experiments of the cells incubation with sRL2 with and without addition of NaN_3 SI9-SI10

Experiment with brief cells incubation with sRL2. Exposure to trypsin SI11

Statistics calculation SI12

Confocal microscopy SI13

Two components simulation SI14-16

MNQKQPACHE	NDERPFYQKT	APYVPMYYVP	NSYPYYGTNL	YQRRPAIAIN
NPYVPRTYYA	NPAVVRPHAQ	IPQRQYLPNS	HPPTVVRRPN	LHPSFIAIPP
KKIQDKIIIP	TIGGSHHHHH	H		

Table S1. RL2 sequence in single letter designation. Highlighted letters: (green) – lysine, (blue) – cysteine.

Spin labeling of RL2

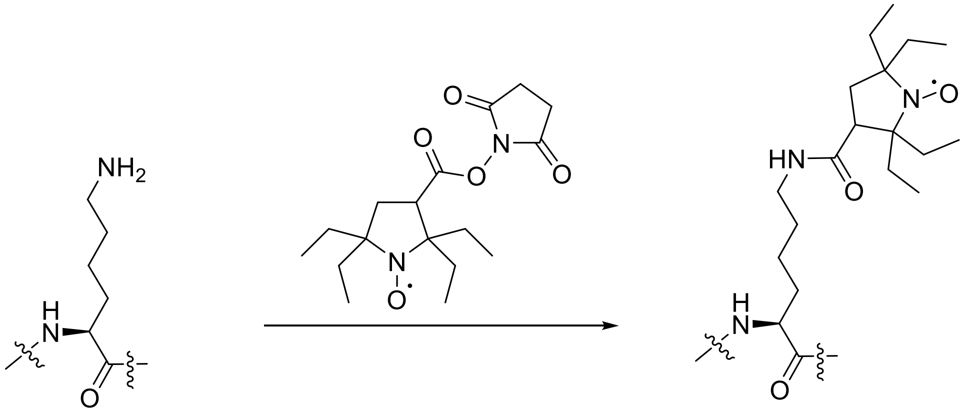

Fig. S1. Labeling of lysine residue using spin label **1**.

We performed several procedures of spin labeling of $RL2_2$ to obtain the samples of sRL2 with different molar ratios of spin label 1 to $RL2_2$. We observed that at high values of the molar ratios (up to 10) the EPR spectrum of the sample contains broadened part (see Fig. S3), while for the samples with the ratios equal to 4 or less the EPR spectra shape was reproducible. When A549 cells were incubated with the sample of sRL2 with the molar ratio 8.7 we observed significant broadening of the EPR spectra of the cells after their incubation (see Fig. S4). The spectrum (Fig. S4, red) cannot be well simulated by three spectral components.

Fig S2. SDS-PAGE: first column - marker 4-20% TRIS-glycine SDS-PAGE; second column clean RL2 dimer ($RL2_2$).

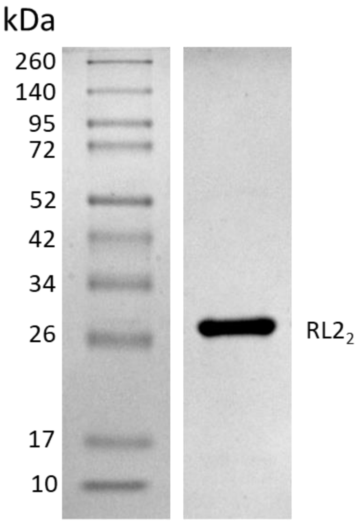

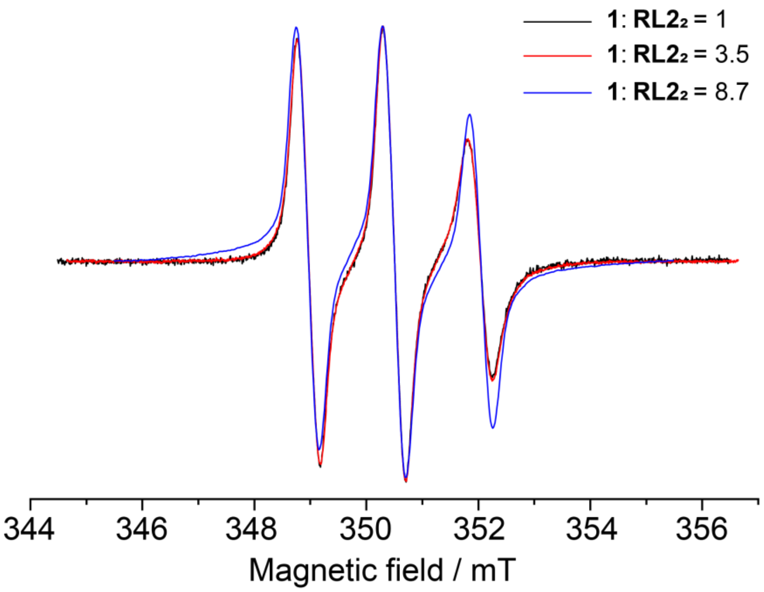

Fig. S3. EPR spectra of the samples of sRL2 in aqueous solution with different molar ratios of spin label 1 to $RL2_2$. The spectra are normalized to their central signal height. Black spectrum corresponds to the sample of sRL2 ($RL2_2$ concentration 0.3 mM) with the ratio of spin label 1 to $RL2_2$:1. Red – the sample of sRL2 ($RL2_2$ concentration 0.16 mM) with the ratio of spin label 1 to $RL2_2$:3.5. Blue – the sample of sRL2 ($RL2_2$ concentration ~0.3 mM) with the ratio of spin label 1 to $RL2_2$:8.7 containing a portion of unwashed spin label 1.

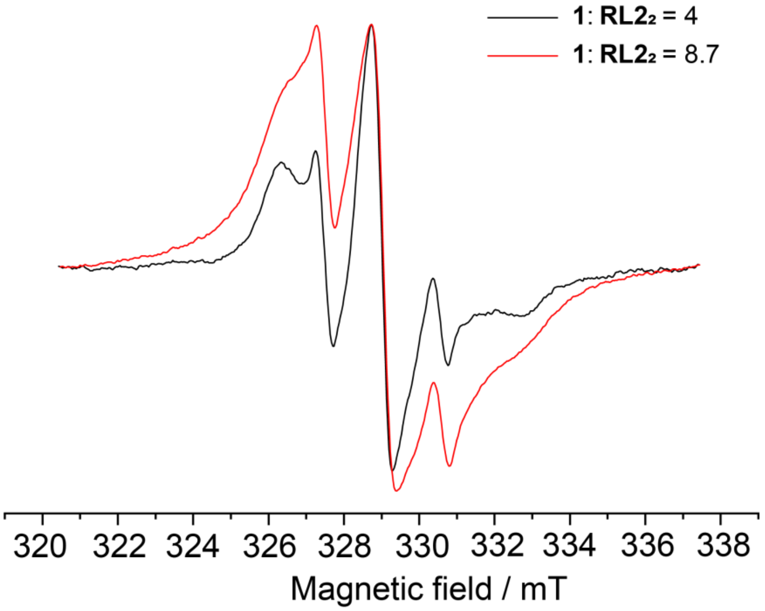

Fig. S4. EPR spectra of A549 cells incubated with sRL2 samples with molar ratios of spin label **1** to $RL2_2$: 4 (black) and 8.7 (red). The spectra were acquired in 1 hour after the end of incubation of cells with sRL2 samples. The spectra presented are normalized to their central signal height.

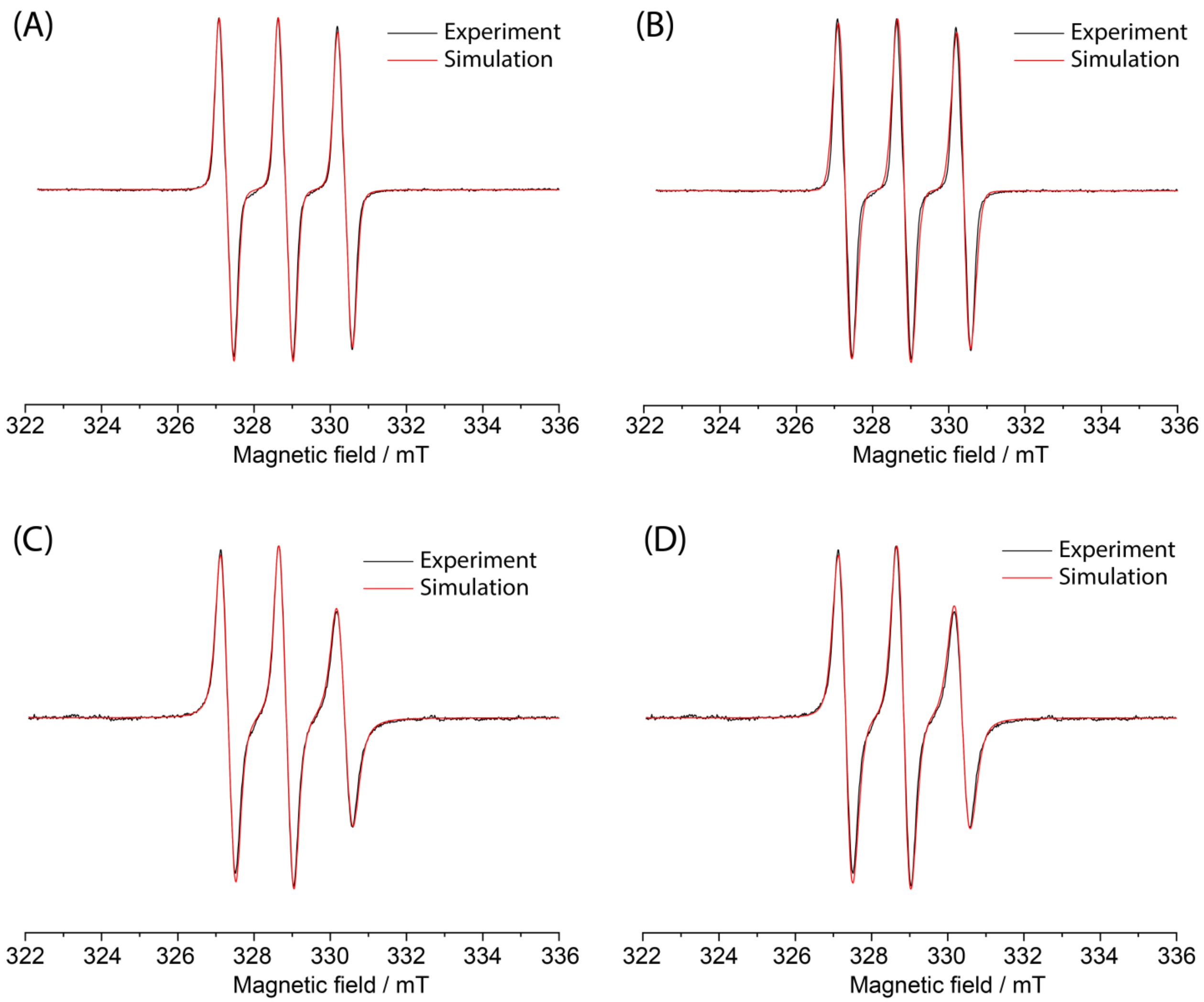

Fig S5. Black – experimental EPR spectra of radical **2** (A, B) in 50 mM phosphate-buffered saline and sRL2 (C, D) (molar ratio of spin label 1 to $RL2_2$: 3.5) in an aqueous solution. Red – simulation of experimental spectra with (A, C) and without (B, D) utilizing A_H parameter in simulation. Spectra (A, C) were calculated with following parameters: (A) A_N = (0.30 0.30 4.08) (mT), A_H = (0.193 236 0.193 0.193) (mT) and (C) A_N = (0.30 0.30 4.00) (mT), A_H = (0.193 0.193 0.193) (mT); line width: (A) (Gaussian 0.223, Lorentzian 0.032) (mT), (C) (Gaussian 0.187, Lorentzian 0.081) (mT); Correlation times: (A) $\tau_c = 4.1 \times 10^{-2}$ ns, (C) $\tau_c = 2.78 \times 10^{-1}$ ns.

Spectra (B, D) are simulated without A_H parameter but with increased line width values. The simulation parameters are (B) A_N = (0.30 0.30 4.08) (mT) and (D) A_N = (0.30 0.30 4.00) (mT); line width: (B) (Gaussian 0.346, Lorentzian 0.0019) (mT) and (D) (Gaussian 0.318, Lorentzian 0.042); Correlation times: (B) $\tau_c = 4.0 \times 10^{-2}$ ns, (D) $\tau_c = 2.77 \times 10^{-1}$ ns.

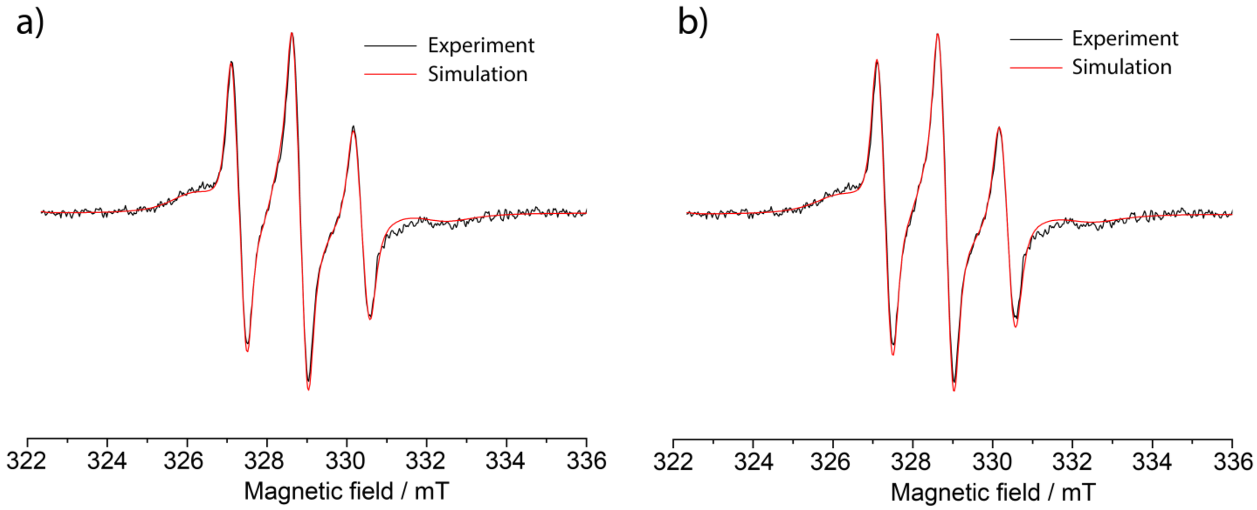

Fig S6. Black – experimental EPR spectrum of A549 cells after their incubation with 1.34 μM sRL2 (molar ratio of spin label 1 to $RL2_2$: 3.5). The spectrum was acquired at 1.02 h from the end of the cell incubation. Red – simulation of the experimental spectrum with (a) and without (b) utilizing A_H parameter in simulation. The simulation parameters of the three spectral components are as follows: $A_1 = A_2 = A_3 = [A_N = (0.30\ 0.30\ 4.08)$ (mT), $A_H = (0.193\ 0.193\ 0.193)$ (mT)]; Correlation times: τ_c1 = 4.77 ns, $\tau_{c2} = 2.95 \times 10^{-1}$ ns, and $\tau_{c3} = 4.1 \times 10^{-2}$ ns; The second integral weight ratios component(1): component(2): component(3) = 0.69:0.28:0.03.

Spectrum (b) is obtained without A_H parameter in simulation. The simulation parameters of the three spectral components are as follows $A_1 = A_2 = A_3 = [A_N = (0.30\ 0.30\ 4.08)$ (mT)]. Correlation times: τ_{c1} = 4.77 ns, $\tau_{c2} = 2.95 \times 10^{-1}$ ns, $\tau_{c3} = 4.1 \times 10^{-2}$ ns; The second integral weight ratios component(1): component(2): component(3) = 0.69:0.28:0.03.

It can be seen that simulations without the constant A_H give a good simulated spectrum in almost all situations. For radical 2 in water, taking into account the constant A_H gives better modeling. However, kinetics of the experimental spectra of spin labeled sRL2 are well modeled with the same weights in both cases. The obtained weights of forms 1, 2, 3 do not change due to the inclusion of A_H.

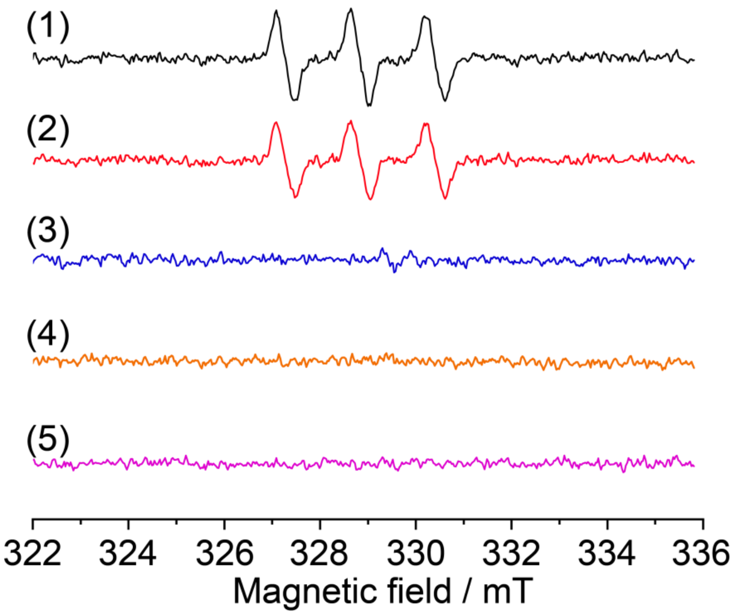

Fig. S7. EPR spectra of **(**1-2**)** cell medium with dissolved nitroxide **2** before (1) and after (2) cells incubation; (3-5) solutions applied to wash the cells in order as presented (3) – PBS, (4) – trypsin, (5) – PBS. The external magnetic field was modulated with a frequency of 100 kHz and amplitude of 2 G. The microwave power was 2.0 mW and all measurements were conducted at room temperature with a time constant of 20.48 ms, conversion time 19.55 ms, sweep time 10.01 s, 512 points, 128 scans.

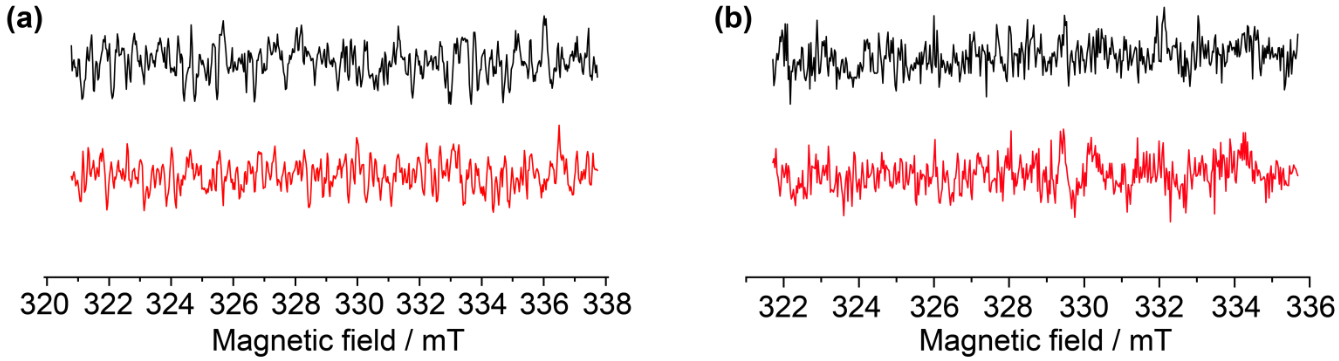

Fig. S8. CW EPR spectra of the cell medium with dissolved sRL2 (molar ratio of spin label **1** to $RL2_2$ – 4) before (black) and after (red) cells incubation in case of high, 5.5 µM (**a**), and low, 0.5 µM (**b**), sRL2 concentrations. The external magnetic field was modulated with a frequency of 100 kHz and amplitude of 2 G. The spectra were acquired at room temperature with the microwave power 2.0 mW. The EPR spectra parameters were as follows: (**a**) time constant 40.96 ms, conversion time 30.91 ms, sweep time 15.83 s, 512 points, 32 scans and (**b**) time constant 20.48 ms, conversion time 58.61 ms, sweep time 30.01 s, 512 points, 48 scans.

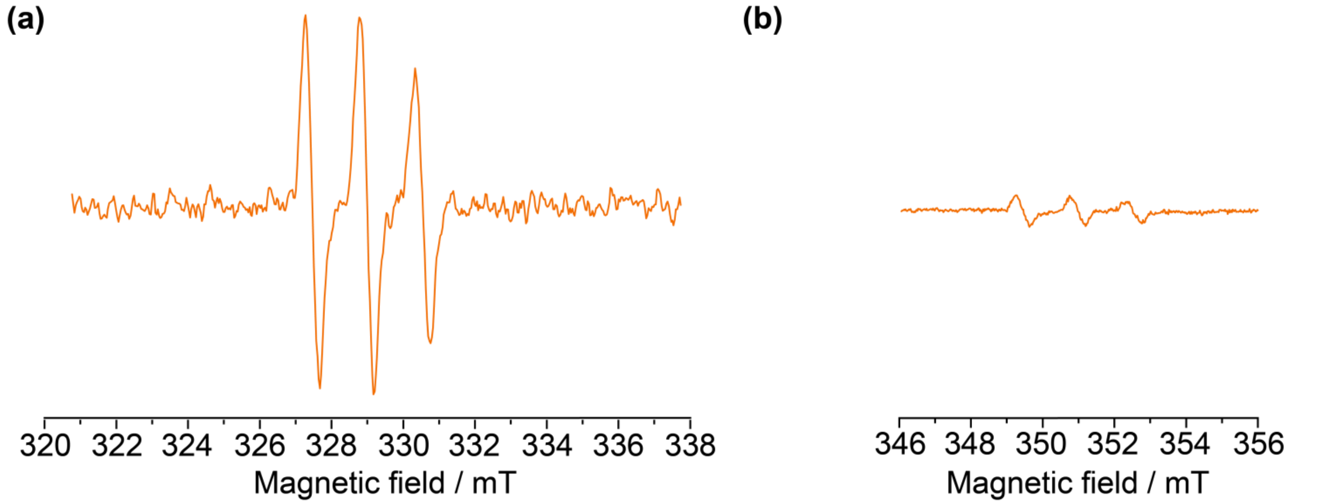

Fig. S9. CW EPR spectra of trypsin solutions after their application to wash the cells incubated with high, 5.5 µM (**a**), and low, 0.5 µM (**b**), sRL2 concentrations. The spectra are presented in absolute scale. The values of apparent spin concentration are (**a**) 4 µM, (**b**) 0.7 µM in 2 ml volume of trypsin solution. The external magnetic field was modulated with a frequency of 100 kHz and amplitude of 2 G. The spectra were acquired at room temperature at microwave frequency (**a**) 9.230426 GHz, (**b**) 9.227356 GHz, with the microwave power 2.0 mW. The EPR spectra parameters were as follows: (**a**) time constant 40.96 ms, conversion time 30.91 ms, sweep time 15.83 s, 512 points, 32 scans and (**b**) time constant 20.48 ms, conversion time 29.30 ms, sweep time 15.00 s, 512 points, 4096 scans.

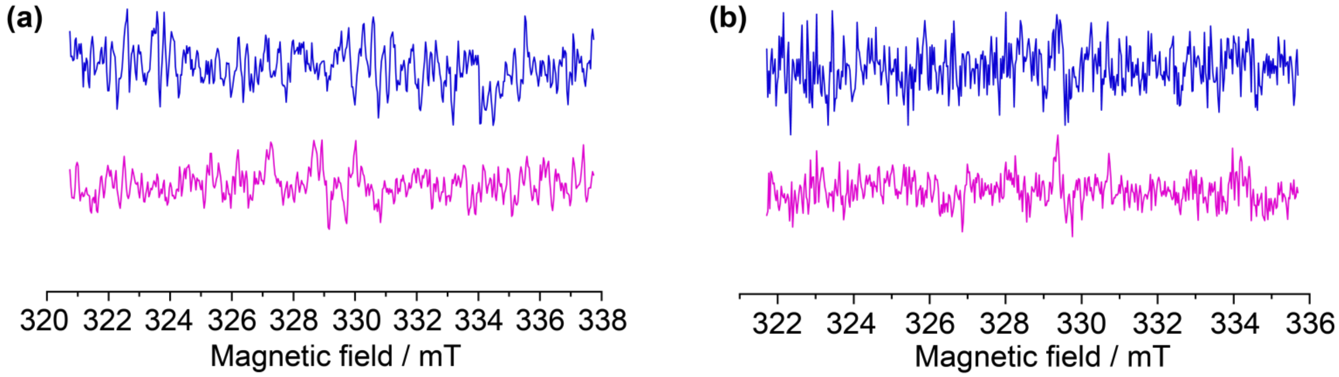

Fig. S10. CW EPR spectra of PBS solutions after their application to wash the cells before (blue) and after (magenta) cells washing by trypsin in case of high, 5.5 μM (**a**), and low, 0.5 μM (**b**), sRL2 concentrations. Volumes of PBS solutions are (**a**) 8 ml (blue) and 2.5 ml (magenta); (**b**) 12.7 ml (blue) and 3 ml (magenta). The external magnetic field was modulated with a frequency of 100 kHz and amplitude of 2 G. The spectra were acquired at room temperature with the microwave power 2.0 mW. The EPR spectra parameters were as follows: (**a**) time constant 40.96 ms, conversion time 30.91 ms, sweep time 15.83 s, 512 points, 32 scans and (**b**) time constant 20.48 ms, conversion time 58.61 ms, sweep time 30.01 s, 512 points, 48 scans.

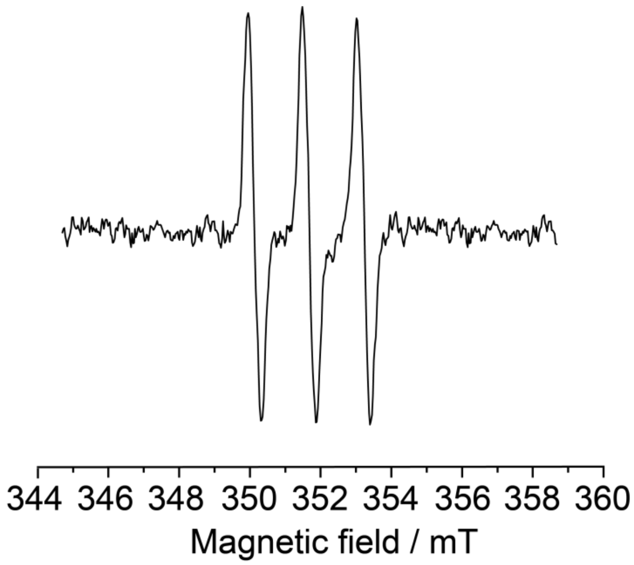

Fig. S11. CW EPR spectrum of the cell medium with dissolved sRL2, 5.5 μM, after addition of large excess of HCl. The external magnetic field was modulated with a frequency of 100 kHz and amplitude of 2 G. The spectrum was acquired at room temperature with the microwave power 2.0 mW. The EPR spectrum parameters were as follows: time constant 20.48 ms, conversion time 19.54 ms, sweep time 10.00 s, 512 points, 8 scans.

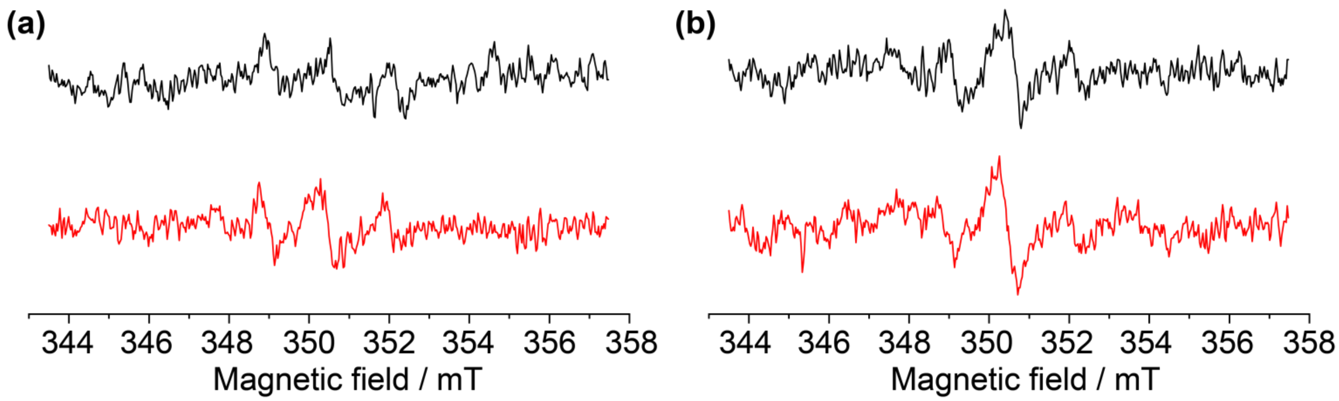

Fig. S12. CW EPR spectra of the cell medium with dissolved 1.34 μM sRL2 (molar ratio of spin label **1** to $RL2_2$ – 3.5) without (**a**) and with (**b**) addition of 1% NaN_3 before (black) and after (red) cells incubation. The spectra are presented in absolute scale. The external magnetic field was modulated with a frequency of 100 kHz and amplitude of 2 G. The spectra were acquired at room temperature with the microwave power 2.0 mW. The EPR spectra parameters were as follows: (**a**) time constant 20.48 ms, conversion time 19.57 ms, sweep time 10.02 s, 512 points, 2048 scans and (**b**) time constant 20.48 ms, conversion time 19.59 ms, sweep time 10.03 s, 512 points, 2048 scans.

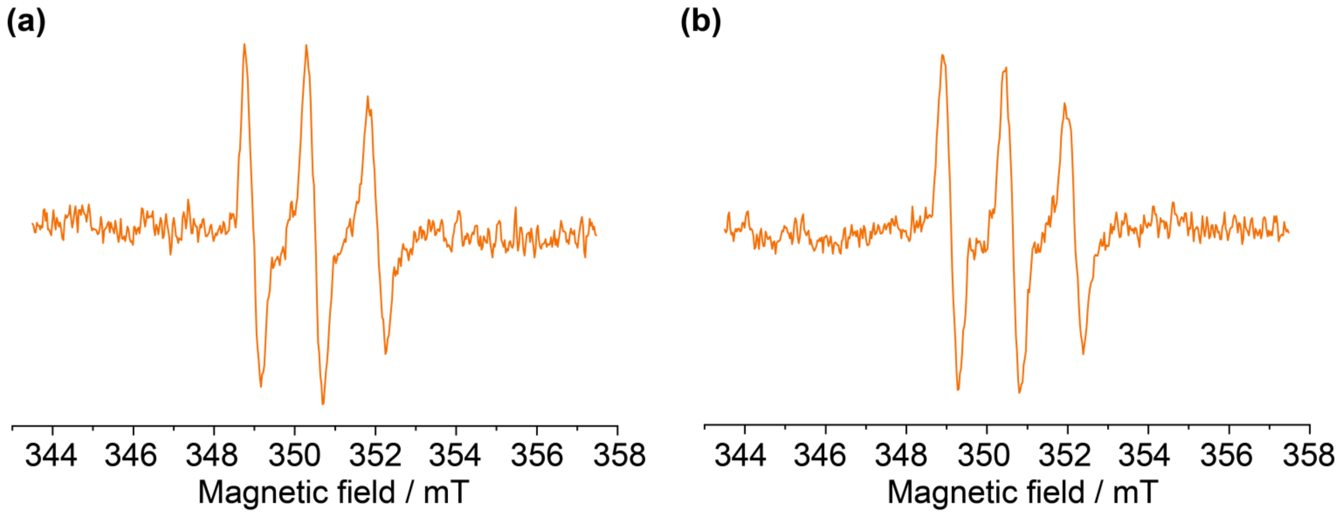

Fig. S13. CW EPR spectra of trypsin solutions after their application to wash the cells incubated in the medium with dissolved 1.34 μM sRL2 (molar ratio of spin label **1** to $RL2_2$ – 3.5) without (**a**) and with (**b**) addition of 1% NaN_3. The spectra are presented in absolute scale. The external magnetic field was modulated with a frequency of 100 kHz and amplitude of 2 G. The spectra were acquired at room temperature with the microwave power 2.0 mW. The EPR spectra parameters were as follows: (**a**) time constant 20.48 ms, conversion time 19.56 ms, sweep time 10.01 s, 512 points, 1024 scans and (**b**) time constant 20.48 ms, conversion time 19.57 ms, sweep time 10.02 s, 512 points, 1024 scans.

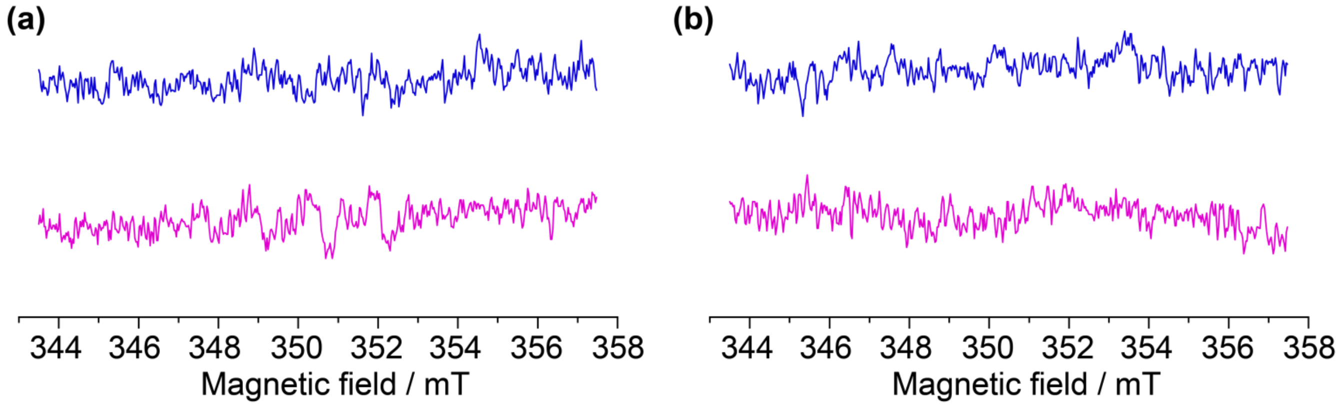

Fig. S14. CW EPR spectra of PBS solutions after their application to wash the cells incubated with 1.34 μM sRL2 (molar ratio of spin label **1** to $RL2_2$ – 3.5) without (**a**) and with (**b**) addition of 1% NaN_3 before (blue) and after (magenta) cells washing by trypsin. The external magnetic field was modulated with a frequency of 100 kHz and amplitude of 2 G. The spectra were acquired at room temperature with the microwave power 2.0 mW. The EPR spectra parameters were as follows: (**a**) time constant 20.48 ms, conversion time 19.57 ms, sweep time 10.02 s, 512 points, 2048 scans and (**b**) time constant 20.48 ms, conversion time 19.59 ms, sweep time 10.03 s, 512 points, 2048 scans.

Brief cells incubation with sRL2. Exposure to trypsin

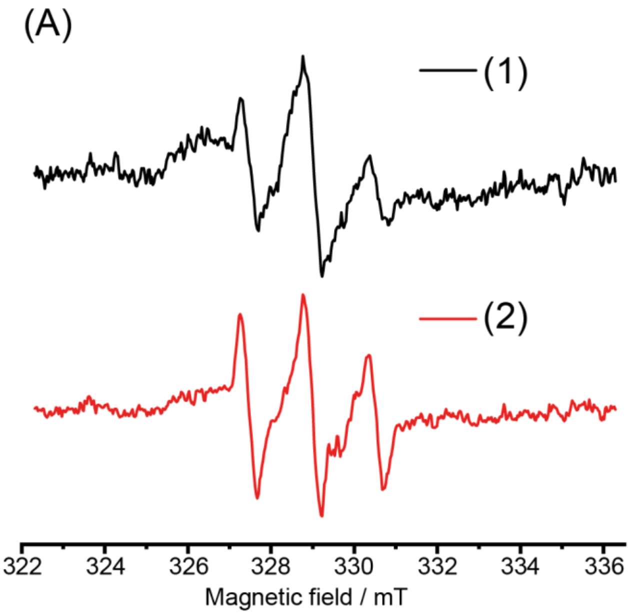

Fig. S15. EPR spectra (A) and the corresponding first integrals (B) of A549 cells incubated during 10 minutes with 1.34 μM sRL2 (molar ratio of spin label **1** to $RL2_2$: 3.5) without (1) and with (2) cells incubation with trypsin. The ratio of the corresponding second integrals for the experiments with and without trypsin incubation is 0.78:1. The spectra were acquired at 35ºC with the following parameters: microwave frequency (1) 9.230606 GHz, (2) 9.229801 GHz; (1, 2) microwave power 2.0 mW; (1, 2) conversion time 19.58 ms; (1, 2) sweep time 10.02 s; (1, 2) time constant 20.48 ms; (1, 2) 512 points; the number of scans (1, 2) 256. In both cases, the external magnetic field was modulated at frequency 100 kHz and modulation amplitude 0.2 mT.

Statistics calculation

In our model we suppose that all Lys positions can be labeled independently and equally probable. The task can be reduced to calculating the probability that two adjacent boxes will be filled with two balls. The numbers 4 and 5 or 9 and 10 are adjacent Lys positions. The molar ratio of spin label 1 to $RL2_2$: 4 is Population mean and can be expressed by the formula

$$4 = w_0 * 0 + w_1 * 1 + w_2 * 2 + \cdots + w_{10} * 10 \quad (1)$$

Where w_i are the probability that i number of positions are labeled. Where

$$\sum_i w_i = 1 \quad (2)$$

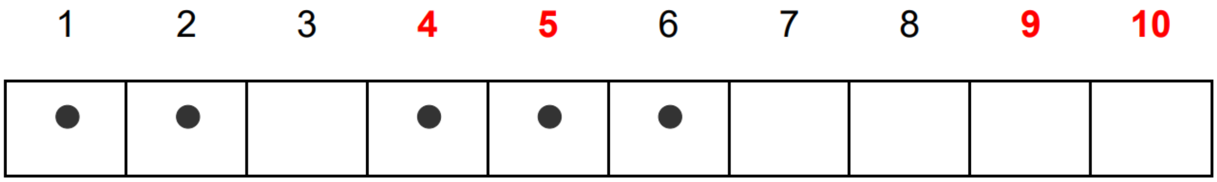

w_i can be calculated by Bernoulli scheme:

$$w_i = C_{10}^i * q^i * (1-q)^{10-i} \quad (3)$$

$$\text{where } C_{10}^i = \frac{10!}{i! * (10-i)!} \quad (4)$$

where q^i is the probability of “success” to find i number of labels in the dimer, $(1 - q)^{10-i}$ is the probability of “failure” to find the remaining (10 - i) number of labels in the same dimer.

If we put (3, 4) in (1), we see that q = 0.4

Then, for all dimers with spin labels in positions (4, 5), (9, 10), (4, 5, 9, 10), the total intensity of 101Lys and 102Lys in the EPR spectra will be:

$$I = 2 * [2q^2(1-q)^8 + 16q^3(1-q)^7 + (2C_8^2 - 1)q^4(1-q)^6 + (2C_8^3 - 6)q^5(1-q)^5 +$$

$$+(2C_8^4 - C_6^2)q^6(1-q)^4 + (2C_8^5 - C_6^3)q^7(1-q)^3 + (2C_8^5 - C_6^3)q^7(1-q)^3$$

$$+ (2C_8^6 - C_6^4)q^8(1-q)^2 + 4q^9(1-q)^1] +$$

$+4 * [q^4(1-q)^6 + 6q^5(1-q)^5 + C_6^2 q^6(1-q)^4 + C_6^3 q^7(1-q)^3 + C_6^4 q^8(1-q)^2 + 6q^9(1-q)^1 + q^{10}]$ = 0.689102848 that is ~ 17% of 4 – the total intensity.

Confocal microscopy

Time, h	Lysosome number	RL2 object number	Colocalization, %
0	73.0 ± 24.4	27.0 ± 14.8	37.0 ± 10.0
3	104.5 ± 56.3	36.5 ± 6.8	40.7 ± 2.9
6	72.0 ± 25.4	29.5 ± 13.8	41.8 ± 18.7
24	64.5 ± 41.3	45.5 ± 8.6	27.7 ± 15.2

Table S2. Colocalization analysis of green and red signals inside A549 cells.

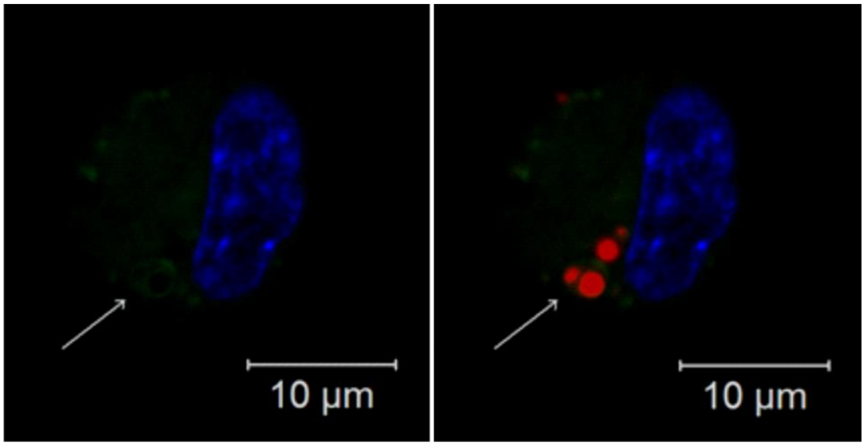

Fig. S16. Localization of the fluorescent RL2 conjugate in A549 cells in 6 hours after their incubation. Regions of the intranuclear localization of Hoechst 33342 are blue, regions of RL2 localization are red, lysosomes and endosomes with pH below 5.5 are green. The arrow marks an autophosome.

Two components simulation

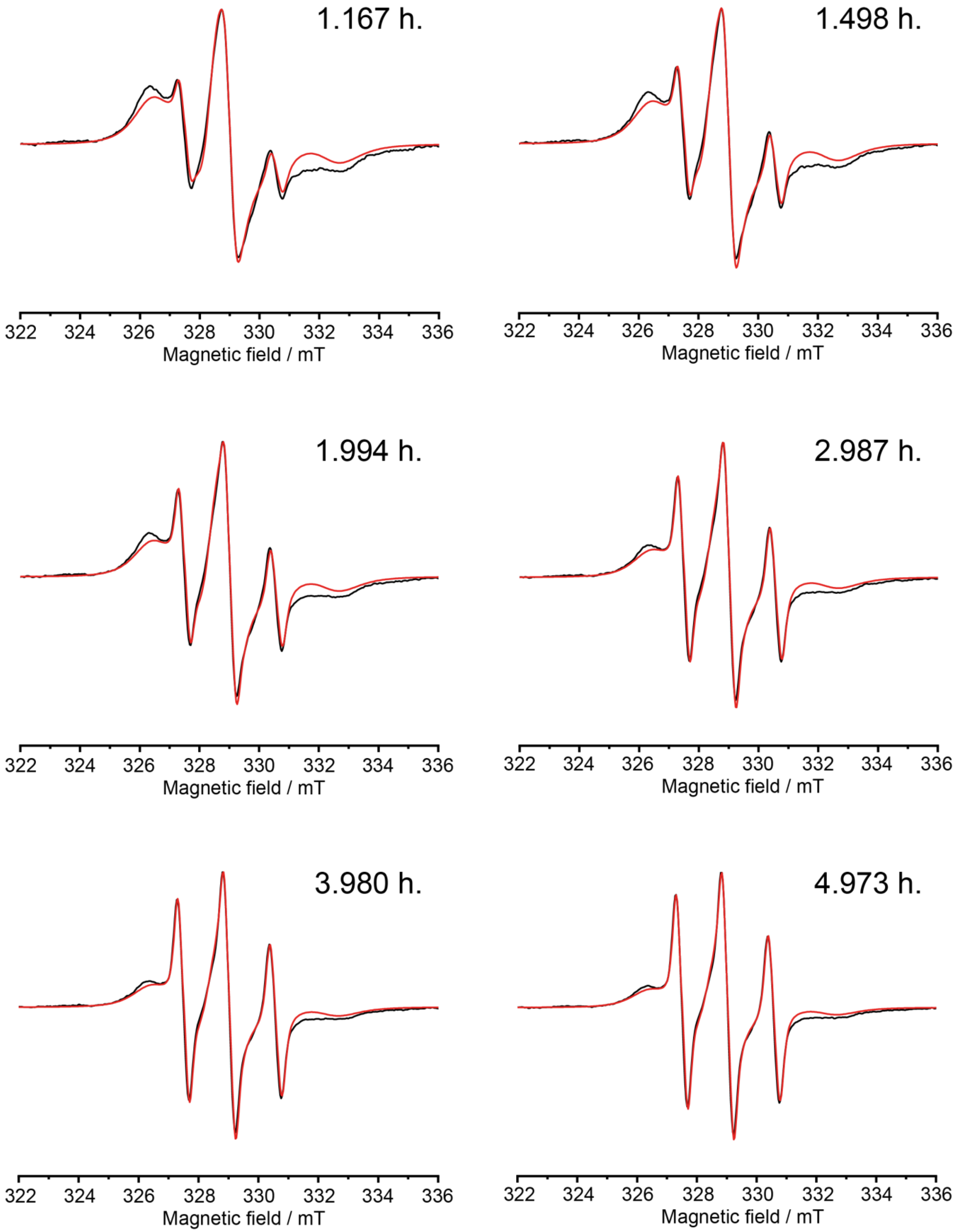

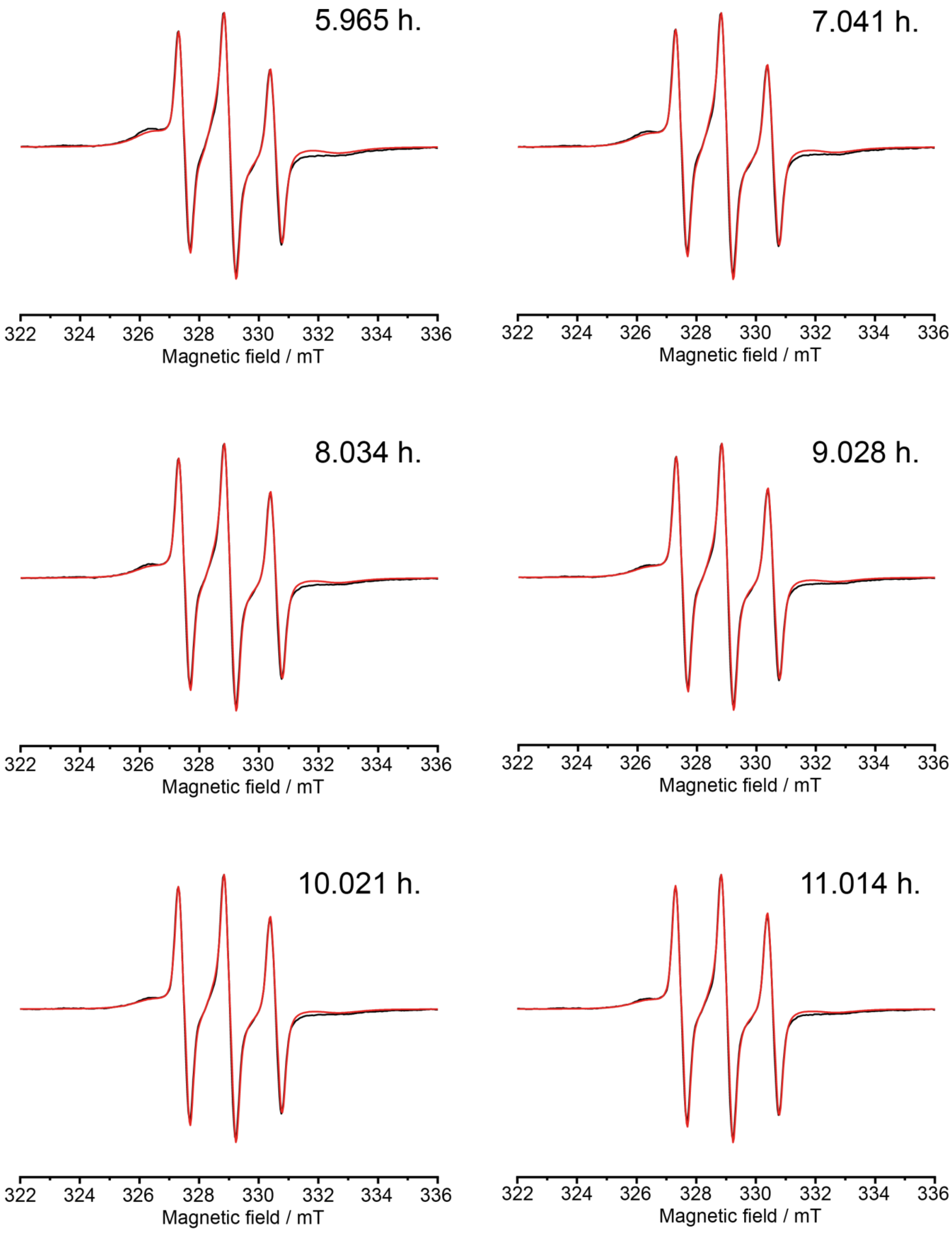
5.965 h.
7.041 h.
8.034 h.
9.028 h.
10.021 h.
11.014 h.
322 324 326 328 330 332 334 336
Magnetic field / mT

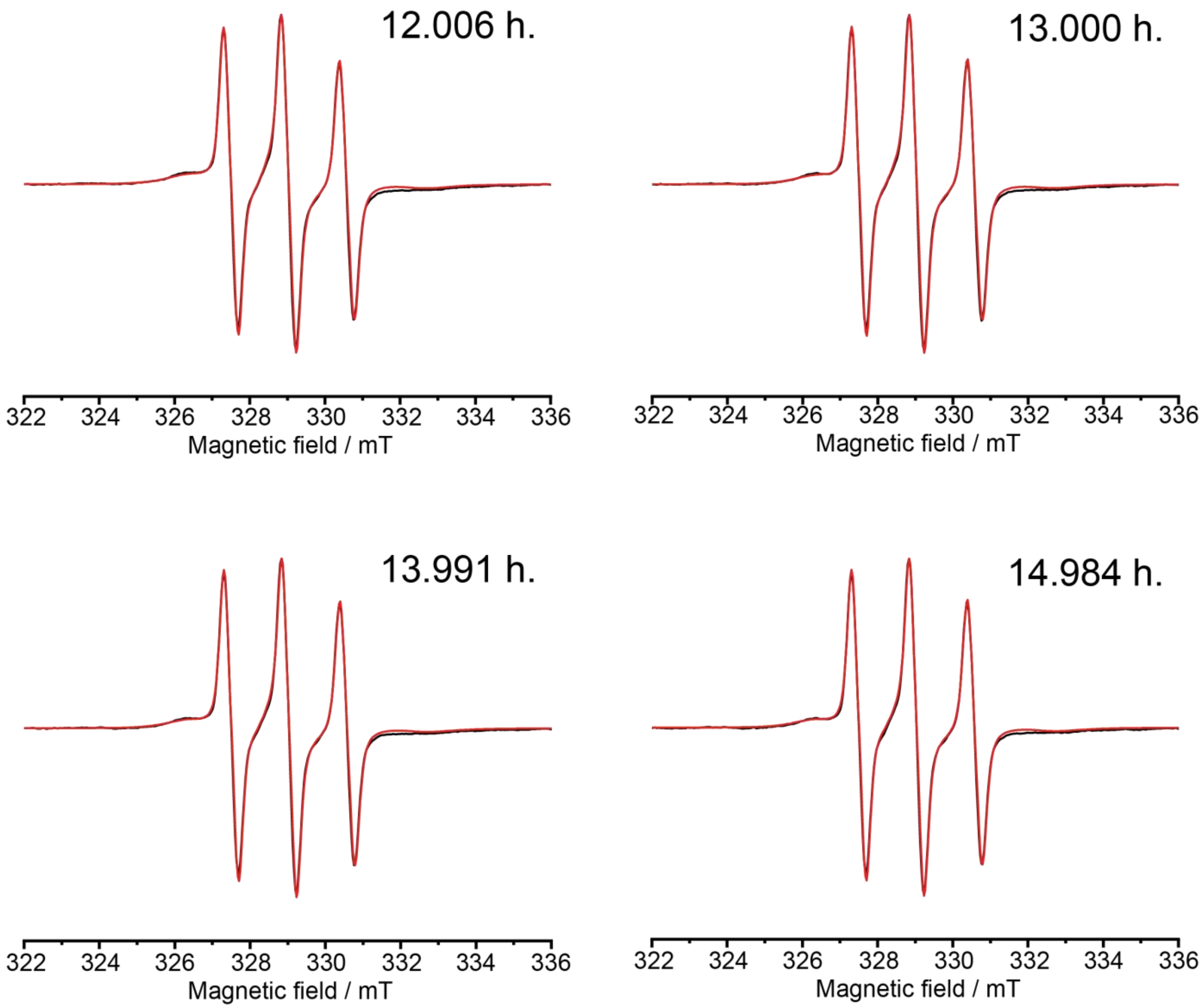

Fig. S17. Black: experimental EPR spectra of A549 cells after their incubation with 5.5 µM sRL2 (molar ratio of spin label **1** to $RL2_2$: 4). Signature of time in hours for each spectrum denotes the acquisition time from the end of the cell incubation. Red: simulation of the experimental spectra by only two components (low mobile component 1* and highly mobile component 2*) with the following simulation parameters: g1* = g2* = [2.0091 2.0059 2.0018]; A1* = [0.30 0.30 3.9], mT, A2* =.[0.30 0.30 4.05], mT. The dependence of the rotation correlation times of the components and the individual component second-integral decay kinetics are presented in Fig 8(a, b) in the article.
